# Yb^3+^-Doped GaN Nanoceramics as a New Material for Broad Band White Light Emission

**DOI:** 10.1021/acs.jpcc.6c00934

**Published:** 2026-04-08

**Authors:** Agata Musialek, Robert Tomala, Giorgio Enrico Gagliardo Briuccia, Wieslaw Strek

**Affiliations:** 215275 Institute of Low Temperature and Structure Research, Polish Academy of Sciences, Wroclaw 50422, Poland

## Abstract

The effect of grain boundary defects on the optoelectronic properties of Yb^3+^-doped GaN nanoceramics was investigated. Structural analysis confirmed crystallization in the wurtzite structure, with a maximum concentration of Yb^3+^ up to 3%. Optical spectroscopy in the visible range allowed us to confirm the defect nature of the obtained materials, and VUV measurements confirmed the presence of deep surface defects, which are active recombination centers for broad band white light emission. In the NIR range, the characteristic emission of the f–f Yb^3+^ transitions was confirmed, as well as energy transfer between the Yb^3+^ ions and the host. Laser-induced white emission (LIWE) was observed after irradiation with CW NIR laser diode in vacuum, revealing the excitation threshold and nonlinearity with increasing laser power density. The emission process was accompanied by efficient photoconductivity. The defined hysteresis contrast reaches a maximum of over 65% for undoped GaN, while the introduction of Yb^3+^ ions reduces the hysteresis, or optical capacitance, reaching a minimum value of almost 23% for the 3% Yb^3+^ sample. The obtained results clearly demonstrate that the obtained nanoceramics can behave as optically controllable memristive-like systems.

## Graphical abstract

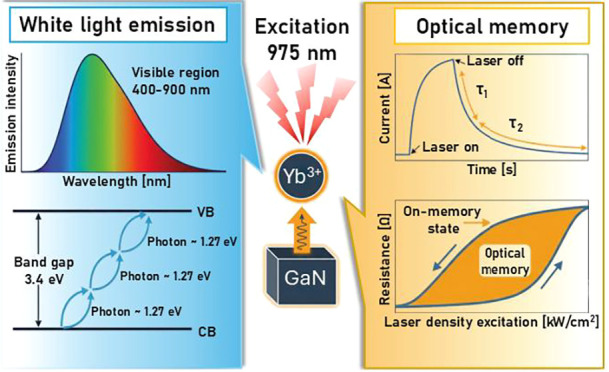

## Introduction

In recent years, III–V semiconductor materials have attracted growing interest due to their chemical and physical properties, such as a wide band gap and high thermal stability. These properties make them suitable for electronic and optoelectronic applications.^1,2^ Gallium nitride (GaN), with an energy gap of 3.4 eV,^3^ is a representative example of this group of semiconductors. It has become increasingly important due to its potential applications in modern LEDs, lasers, detectors, and transistors.^2^ Its band gap value is three times higher than that of Si-based materials, enabling GaN devices to operate at higher temperatures and voltages. GaN-based materials are characterized by high thermal conductivity, which improves heat dissipation in high-power devices. To enhance luminescent properties, rare earth ions are often used as dopants.^4,5^ Single-crystalline GaN layers deposited on substrates, such as sapphire, are currently the industrial standard. However, there is growing interest in ceramics and nanocrystalline forms of GaN. Unlike “perfect” thin films, nanoceramics exhibit a high density of grain boundaries. Structural defects are traditionally seen as harmful to the performance of subsequent devices. However, defect engineering is rapidly developing, changing the perception of defects into an advantage where grain boundaries can serve as active functional regions, especially rare earth ions incorporation.^6^ Using lanthanides as impurities in GaN modifies its electronic structure. Doping creates a core consisting of 4f-electrons, which is shielded by the outer 5s^2^ and 5p^6^ electron shells. Due to this shielding, the 4f–4f transitions within the electron shell result in very narrow optical emission lines in the UV-IR range. Charge transfer (CT) or 5d–4f transitions can also occur in these materials.^5^ The most commonly used dopants in GaN are europium and erbium ions, allowing for the design of materials with emission in the visible range. Consequently, GaN:Er (green emission) and GaN:Eu (red emission) can be used in LEDs to generate white light.^7–10^ Many other rare earth ions can also be utilized. One of them, possessing a simple energy level scheme, is the Yb^3+^ ion. It has only two main states: ^2^F_7/2_ and ^2^F_5/2_. The transition to the excited state corresponds to emission in the near-infrared at a wavelength of about 980 nm.^4,6^ Yb^3+^ ions provide an additional characteristic band, and such materials are mainly used in LEDs and NIR lasers. Combining aspects such as the possibility of using RE-doped GaN, luminescence in the visible range, and white light diode design, recent years have seen an intensified search for emission sources that can generate light as close as possible to sunlight. Moreover, in the case of GaN, incorporating RE ions into the wurtzite structure is a challenge and is often limited by low level of incorporation.^1,11^ Among various optical phenomena observed in RE-doped nanomaterials, laser-induced white emission (LIWE) driven by near-infrared excitation has been heavily explored recently.^12^ This phenomenon eliminates the need for traditional phosphors. LIWE has been investigated in transition-metal-doped yttrium aluminum garnets,^13,14^ perovskites,^15^ Sr_2_CeO_4_,^16^ Y_2_Si_2_O_7_.^17^ However, in semiconductor structures like GaN, LIWE has been reported only once and is not fully understood.^18^ The mechanism of this emission is the subject of debate. Hypotheses suggest that the mechanism may involve electron–hole recombination leading to the formation of oxygen vacancies.^19^ Electrons caught by these vacancies cause emission of the valence band. Another mechanism assumes blackbody radiation,^20^ where the process is related to host matrix heating. A further mechanism can be inter-valence charge transfer (IVCT),^21^ assuming a change of oxidation state (Ln^3+^ → Ln^2+^). The investigation of broad band white light emission, especially in nanometric materials, is interesting due to the significant influence of grain size on optical, electronic, and thermal properties. This can potentially lead to the enhancement or modification of emission, for example, due to a larger surface-to-volume ratio.^22^ Simultaneously, the defects occurring in nanoceramics that enable emission play a crucial role in charge transport. The ability to trap and store charge in deep trap states for extended periods can lead to persistent photoconductivity (PPC).^23^ In the field of promising technologies, such as optical memory, this feature is highly desirable. Devices that exhibit history-dependent conductivity (memristors) can be applied as optoelectronic synapses.^24,25^ Despite such promising effects, achieving control over relaxation dynamics and the magnitude of hysteresis remains a huge challenge. Importantly, we assume that Yb^3+^ ions can create additional multiphoton absorption channels and deep traps at grain boundaries. In this paper, we present spectroscopic and electrical measurements allowed for the functionality of GaN:Yb^3+^ nanoceramics.

## Experimental Section

The GaN:Yb^3+^ nanopowders were obtained by two steps: sol–gel-modified Pechini method^26^ and ammonothermal method.^27,28^ Commercial gallium oxide (Ga_2_O_3_, powder, 99.99%, Thermo Scientific) and ytterbium oxide (Yb_2_O_3_, 99.998%, powder, Stanford Materials Corporation) (0, 0.5, 1, 2, 3, 5% mmol, respectively) powders were dissolved in concentrated nitric acid (HNO_3_, 65%, POCH Basic) to obtain their nitrates and recrystallized four times. Then, monohydrate citric acid (C_6_H_8_O_7_·H_2_O, 99.4%, POCH Basic) was added to the solutions and stirred for 1 h. Next, ethylene glycol (C_2_H_6_O_2_, 96%, Chempur) was added and stirred once again for 1 h. The amounts of citric acid and ethylene glycol were added in a 5-fold excess to the gallium nitrate. The samples were put into a dryer at 90 °C for 1 week until resins formed, which were calcinated at 900 °C for 6 h in an air atmosphere. The second step was calcination in the ammonia flow of the previously obtained nanopowders. The samples were annealed in a tube furnace in an ammonia flow at 950 °C for 5 h. The obtained products were yellow nanocrystals. The ceramics were obtained with the low-temperature high-pressure (LTHP) technique sintering process at 8 GPa and 500 °C.^18^ The XRD measurements were performed to check the purity of the samples by using a PANalytical X’Pert Pro X-ray powder diffractometer with a Cu radiation source. Absorption spectra were obtained with an Agilent CARY 5000 UV–vis–NIR equipped with a Praying Mantis adapter (Harrick). Stokes emission was measured on FLS980 Edinburgh Instruments using a CW laser diodes 360 and 940 nm 4 W (CNI Lasers) as an external source of excitation. Anti-Stokes emission was measured in a vacuum atmosphere (1 × 10^–5^ mbar) using a 975 nm CW laser diode (CNI lasers) and AVS-USB2000 (Avantes) spectrometer for a visible range, Ocean Optics NIRQuest for a near-infrared range. Measurements of photoconductivity were recorded using a Keithley 2400 source meter as a detector. Vacuum ultraviolet (VUV) measurements under synchrotron radiation excitation were recorded at the PETRA III P66 beamline of the DESY Synchrotron (Hamburg, Germany). The instrument is equipped with a 0.3 m Kymera 328i Andor monochromator with an F/4.1 aperture (200-1200 nm) and 0.5 nm spectral resolution. The monochromator is also equipped with three interchangeable 300 lines mm^–1^ gratings at 300, 500 and 1200 nm optimized for several spectral regions. The emission signal from the sample is gathered and focused on the monochromator by two quartz lenses. One monochromator port is equipped with a CCD camera (Newton 920) and the other with a set of photomultiplier tube (PMT) detectors: Hamamatsu R6358 and R3809U-50. The monochromator for the VUV range is a specially designed device (Pouey mounting F/2.8) without an entrance slit and with variable density of lines of the diffraction grating. Its operating range is from 115 to 320 nm,currently limited by the solar-blind PMT (Hamamatsu R6836). The combination of both monochromators allows great flexibility over a broad range of wavelengths.^200^ The luminescence properties of the samples were investigated at room temperature (300K) and at 12 K. Low temperature measurements were carried out with a KONTI (Cryovac) closed cycle cryostat.

## Results and Discussion

### Structure and Morphology

X-ray diffraction (XRD) measurements were performed to confirm the structure and phase purity of all nanopowder samples. XRD diffraction patterns of pure GaN and GaN samples doped with ytterbium (Yb) at concentrations of 0.5, 1, 2, 3, and 5% are shown in Figure 1a. Measurements were performed in the angular range 2θ from about 10 to 90°, recording the diffraction intensity as a function of angle.

**Figure 1:**
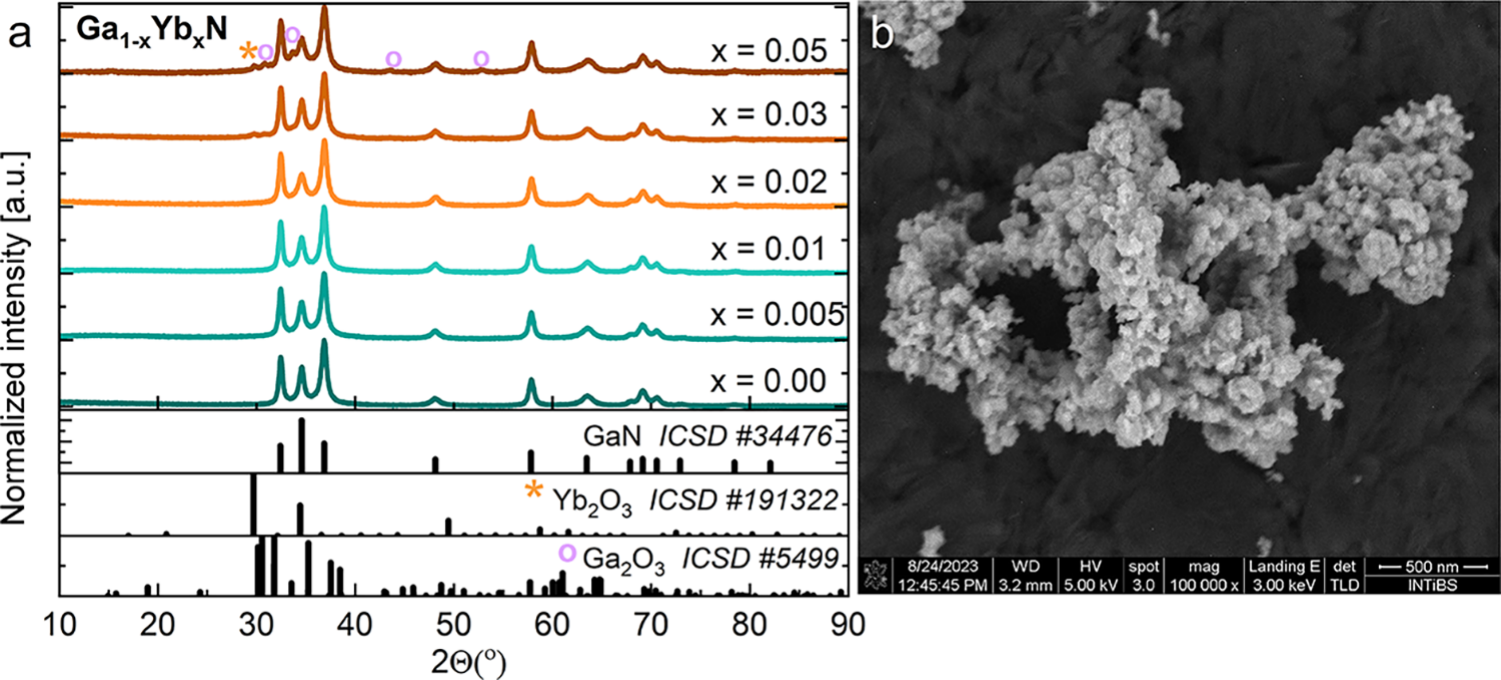
X-ray diffraction patterns (a) and SEM image (b) of Ga_1–*x*_Yb_*x*_N nanopowders.

XRD analysis confirms that all samples are consistent with the pattern for GaN (ICSD #34476). They are characteristic of the wurtzite structure with a hexagonal crystal system and in the *P63mc* space group.^11^ Despite the presence of the dopant, the ordered crystalline structure is preserved. In samples with up to 3% dopant content, all reflections correspond exclusively to GaN. At higher concentrations, two foreign phases appear: one derived from ytterbium oxide (ICSD #191322), which was not incorporated into the gallium nitride due to the size of the ytterbium ions,^6^ and another one derived from gallium oxide (ICSD #5499), because the Ga_2_O_3_ was not completely reacted. The most intense peak for pure GaN corresponds to the (002) plane and occurs at an angle of 2θ = 34.5 degrees. An increase in the dopant concentration also causes a clear decrease in the intensity of the main peak. This is likely the result of a decrease in the degree of crystallinity or an increase in the number of structural defects. A broadening of the diffraction peaks is observed, which indicated the relatively small size of the nanocrystals. Rietveld analysis confirms the crystallite size, which averages 9–13 nm. Therefore, in Figure 1b, the SEM images for the representative sample are shown, and the results for all samples are presented in the Supporting Information (Figure S1). The images confirm the structure of the obtained nanomaterials. The nanoparticles were strongly agglomerated, and their sizes were estimated to be 0.5–2 μm. The calculation of individual nanocrystals is not possible due to the undesirable formation of nanocrystalline clusters.

### Absorption Spectra

The absorption spectra of GaN:Yb^3+^ nanopowders were measured in the visible and near-infrared regions. As shown in Figure 2a, the samples exhibit strong absorption in the visible range originating from GaN.

**Figure 2:**
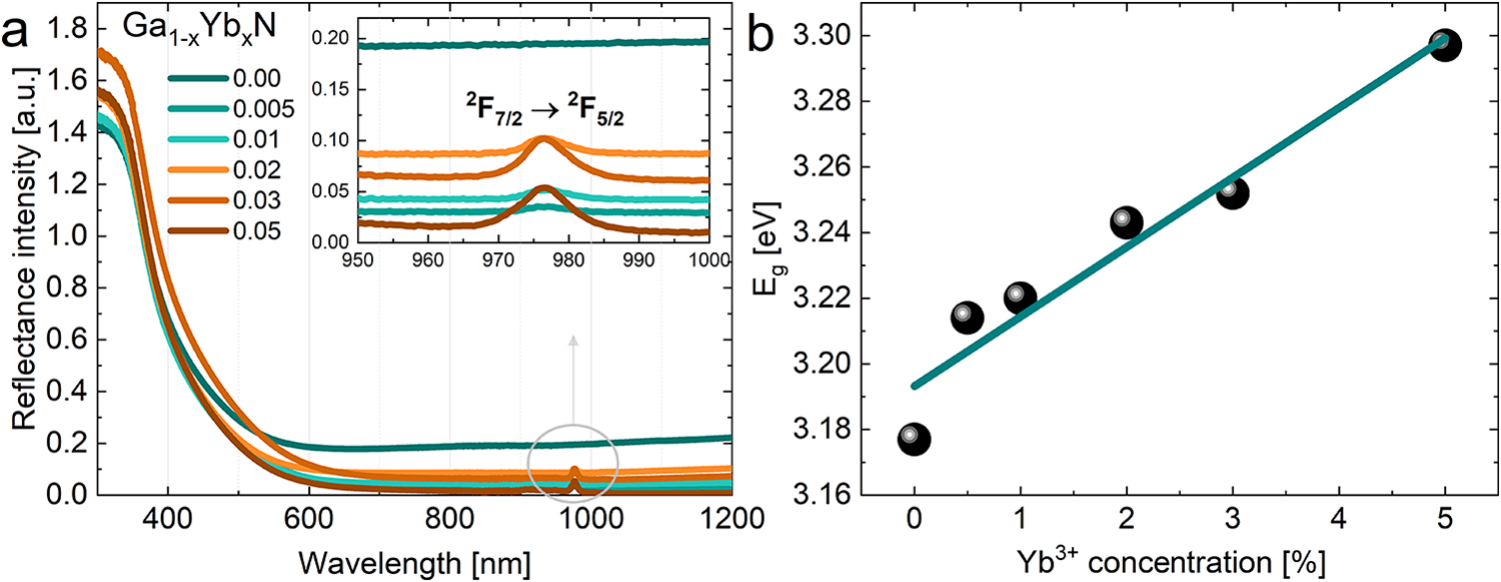
Reflective absorption for GaN:xYb^3+^nanopowders (a) and the dependence of the energy band gap size on dopant concentration (b).

The general trend of absorbance for pure GaN across the entire range shows negligible absorption in the near-infrared and visible regions with a sharp increase occurring in the ultraviolet region. This is typical behavior for semiconductors, especially wide-bandgap ones.^1^ The absorption edge, located around 380 nm, corresponds to a band gap in the range of 3.17–3.30 eV. The observed blue shift of the absorption edge may indicate a change in the band structure with varying dopant content. This edge signifies electron transitions from the valence band to the conduction band, characteristic of GaN. The band gap increases linearly with the concentration of Yb^3+^ dopant (Figure 2b). Based on UV–vis spectra, the band gap values were calculated using the Kubelka–Munk^29^ method and compared with literature values (Figure S2). These values, although slightly lower than the theoretical value of 3.4 eV for pure GaN, are within the range observed for nanocrystalline materials. This suggests that the introduction of Yb^3+^ ions modifies the electronic structure of GaN, affecting the band gap width. Differences in *E*_g_ values compared to those of ideal GaN may result from size effects, structural defects, and interactions between Yb^3+^ ions and the GaN matrix. The probable linear increase in the band gap with increasing dopant concentration is a result of strain in the crystal lattice.^11^ The ionic radius of Yb^3+^ is larger than that of Ga^3+^, leading to a higher strain in the GaN crystal lattice. A key argument is the result for GaN:5% Yb^3+^, where ytterbium ions are not fully incorporated into the matrix.^6^ The sharp and distinct absorption edge observed in the spectrum confirms the crystallographic quality of the obtained materials, which is crucial for their potential optoelectronic applications. On the other hand, slight absorption in the 550–570 nm range may be associated with the occurrence of yellow luminescence, which is commonly attributed to structural defects such as Ga vacancies (V_Ga_).^1,6^ In the near-infrared region, an absorption band is observed at 977 nm, characteristic of the ^2^F_7/2_→^2^F_5/2_ transition of Yb^3+^ ions.^4^ The absorption intensity increases with the concentration of Yb^3+^ ions, confirming their effective incorporation into the GaN structure and their optical activity. The absorption maxima for Yb ions do not show significant shifts as a function of concentration, which may suggest a relatively homogeneous environment for Yb^3+^ ions. The observed yellow color of the materials is caused by the absorption edge covering the blue range. Slight fluctuations in the position of the absorption edge may be related to the induction of strain in the crystal lattice and changes in carrier concentration due to the incorporation of Yb^3+^ ions, while the steepness in the case of doped samples appears to be very similar, indicating that the introduction of Yb^3+^ into the matrix does not lead to significant degradation of the crystal quality in terms of band disturbances.

### Emission Spectra

Figure 3 presents the emission spectra for a series of nanopowder samples with different concentrations of Yb^3+^ ions. The studies include two types of excitations: above the host energy band gap (360 nm) in both the visible (a) and near-infrared regions (b), and resonant intrashell excitation of Yb^3+^ ions (940 nm) (c). Excitation with a 360 nm wavelength leads to the generation of electron–hole pairs between the valence band and the conduction band of the host matrix. Subsequently, the generated carriers can recombine, transferring energy to the luminescent centers. The spectra recorded under 360 nm laser excitation in the visible range are characterized by a broad band from 405 to 650 nm with three overlapping bands with local maxima at 416, 438, and 466 nm.

**Figure 3:**
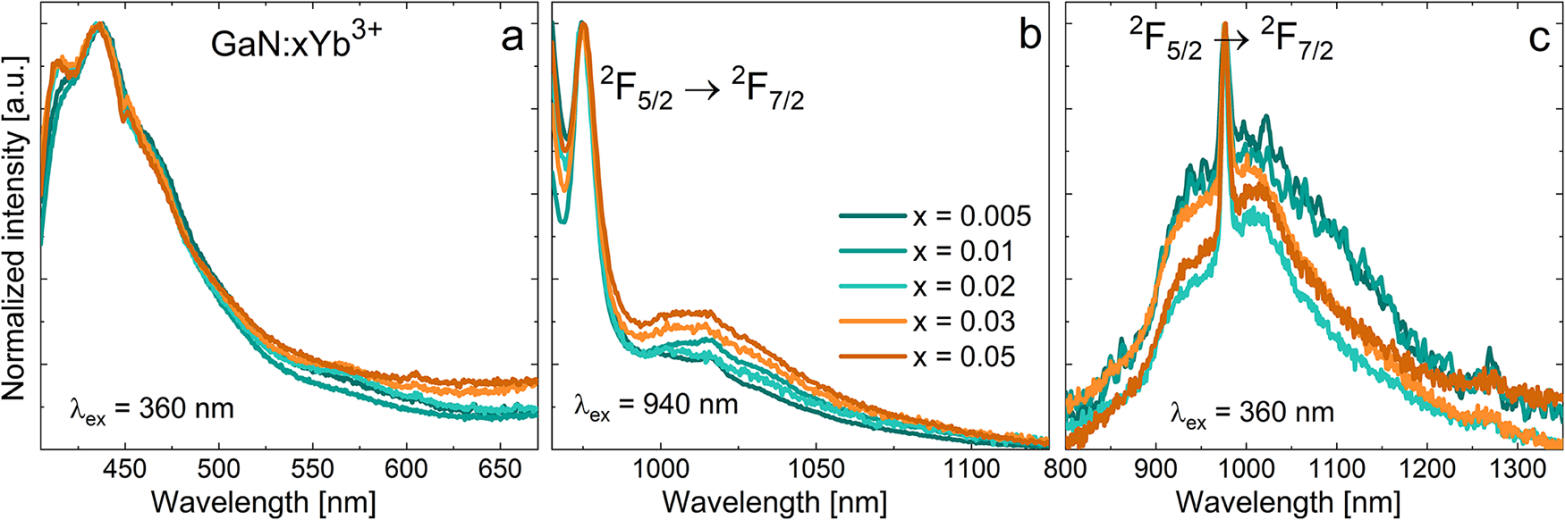
Photoluminescence spectra of Ga_1–*x*_Yb_*x*_N nanopowders at different excitation wavelengths: λ_ex_ = 360 nm in the visible range (a) λ_ex_ = 940 nm (b) and 360 nm (c) in the near-infrared range.

It can be observed that the broad band is shifted toward lower energy compared to the energy gap value for GaN, indicating a redshift.^3^ The presence of this complex band is related to the recombination of carriers at different energy levels associated with point defects. The band with a maximum at 416 nm is typically observed in materials modified by the implantation of additional ions. It is likely associated with structural defects introduced during synthesis and the doping process. This leads to the formation of defects such as V_Ga_ and V_N_ vacancies, which are induced by Yb^3+^ ions.^6^ The band with a local maximum at 438 nm is often referred to in the literature as blue carbon luminescence. It is probably directly related to the presence of carbon atoms at nitrogen positions (C_N_) in the GaN crystal lattice. This defect can act as both an acceptor and a donor. The carbon defect is unintentional but is one of the most common, as organic compounds are used during synthesis.^30^ The band with a local maximum at 466 nm also belongs to blue luminescence. However, its origin is more complex. It is assumed that in this case, recombination occurs on donor–acceptor pairs. Impurities such as oxygen at a nitrogen site (O_N_) can be the donor, and a Ga vacancy can be the acceptor.^3,30^ Deconvolution of the spectrum reveals the complex nature of these defects, allowing us to conclude that the luminescence is caused by mechanisms related to defects, recombination on carbon, and donor–acceptor pairs. A band originating from yellow luminescence is also present in the visible range. It is indicated that this is emission from a carbon defect where carbon substitutes for nitrogen. Furthermore, it is observed that the intensity ratio of the yellow band to the blue band increases with Yb^3+^ concentration.^6^ This means that the yellow band is much less quenched than the blue band. Under 360 nm excitation in the near-infrared range, a characteristic emission for Yb^3+^ ions with a maximum at 975 nm is observed. It is contributed to by the electron transition from the Yb^3+^ excited state ^2^F_5/2_ to the ground state ^2^F_7/2_. The co-occurrence of this emission with excitation above the energy gap is a direct proof of an efficient energy transfer from the GaN host to the Yb^3+^ ions, showing a broad band (800–1250 nm) with a narrow peak at 975 nm.^31–33^ Due to its wide energy band gap, GaN is attractive for doping with ions such as Yb^3+^, because it minimizes the suppression and reabsorption of Yb^3+^ emission and makes the energy transfer more effective.^5^ It is worth noting that in the case of energy transfer to Yb^3+^ exceeding 2%, concentration quenching occurs. The emission spectra recorded under 940 nm excitation, directly into the Yb^3+^ ions, exhibit the characteristic broad band in the range from 968 to 1100 nm with the narrow peak with a maximum at 975 nm. The change of emission intensity with the concentration of Yb ions indicates a typical concentration quenching process. The intensity increases up to a 3% doping level and then decreases, suggesting that the maximum concentration is 3% Yb^3+^.^4^

Luminescence decay curves for nanopowder samples with different Yb^3+^ concentrations are presented in Figure 4. The measurements were performed by using a 940 nm laser diode. The emission was monitored at a wavelength of 975 nm. The decay curves for all samples deviate from a single exponential function. A nonexponential decay is typical for materials in which luminescent centers occupy several different, nonequivalent positions in the crystal lattice.^6^ Based on both emission and absorption, it was determined that defects are present in the obtained materials, causing Yb^3+^ ions to occupy substitutional sites or form complexes with defects. The measured decays are the sum of decays from all of these centers.

**Figure 4:**
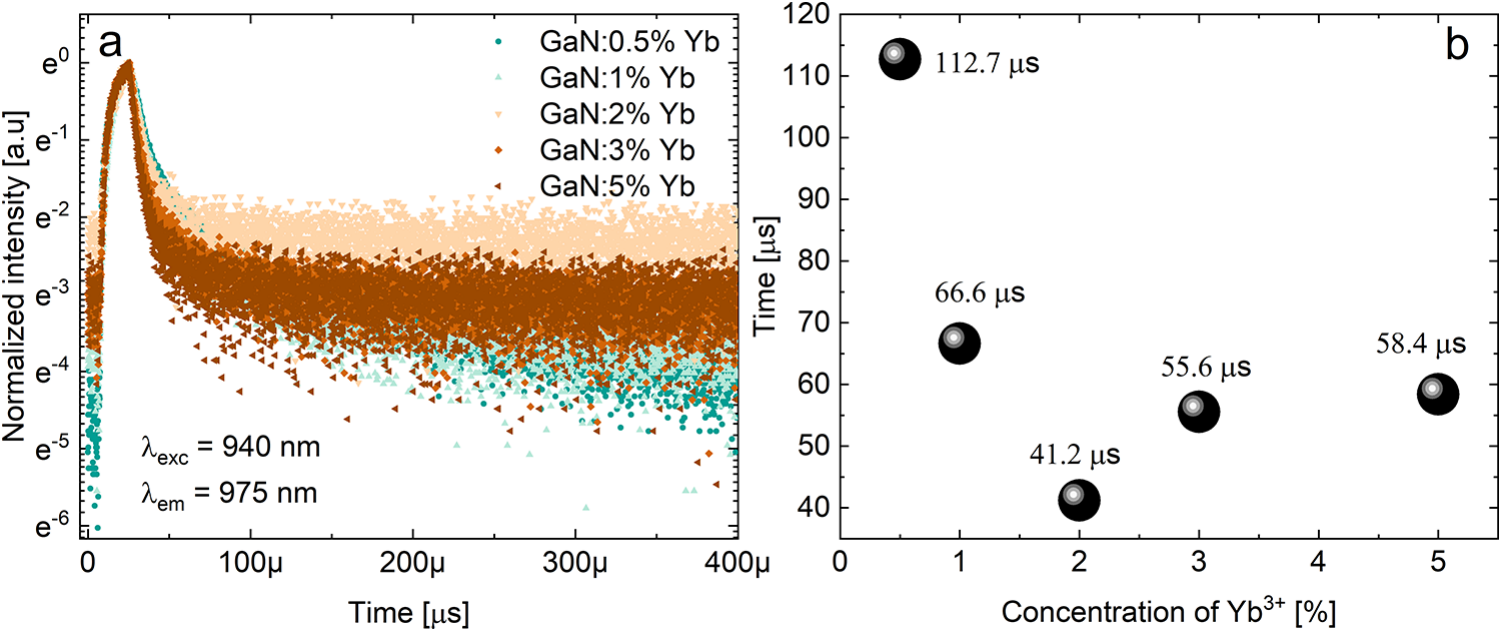
Concentration dependence of emission decay (a) and the concentration dependence of determined decay times of Yb^3+^:GaN nanopowders (b).

The dominant observation is a clear change in the decay rate with an increasing concentration of Yb^3+^ ions. At low dopant concentrations from 0.5 to 2%, a rapid decrease in decay time is observed. This decreasing trend indicates concentration quenching.^4^ However, above 2% Yb^3+^, the decay time increases again. This is probably because, as the dopant concentration increases, processes such as radiation trapping (reabsorption) or the formation of additional clusters with different kinetics begin to play an important role.

Furthermore, to better understand the influence of the defect structure of the obtained nanopowder materials, VUV measurements were performed by exciting materials with a 150 nm beam in high vacuum at 300 and 12 K. The 150 nm excitation corresponds to a region of high absorption coefficient, allowing for the study of nanoparticle surfaces.^34^

In the 350–600 nm range (Figure 5a,d), a typical GaN band originating from internal defects becomes visible, as described in the previous section. Interestingly, these results reveal a band at 700 nm present in Figure 5b,e (red luminescence). During VUV excitation, this luminescence becomes much more readily apparent, which confirms the occurrence of deep defect states, serving as a reservoir for charge traps, which later translates into photoelectric effects in the nanoceramics.^18,23^ In the near-infrared range (Figure 5c,f), emission originates only from f–f transitions of Yb^3+^ ions. Particularly at 12 K, the intraconfigurational f–f emission of Yb^3+^ becomes more pronounced, once again providing evidence for active charge transfer.^5^

**Figure 5:**
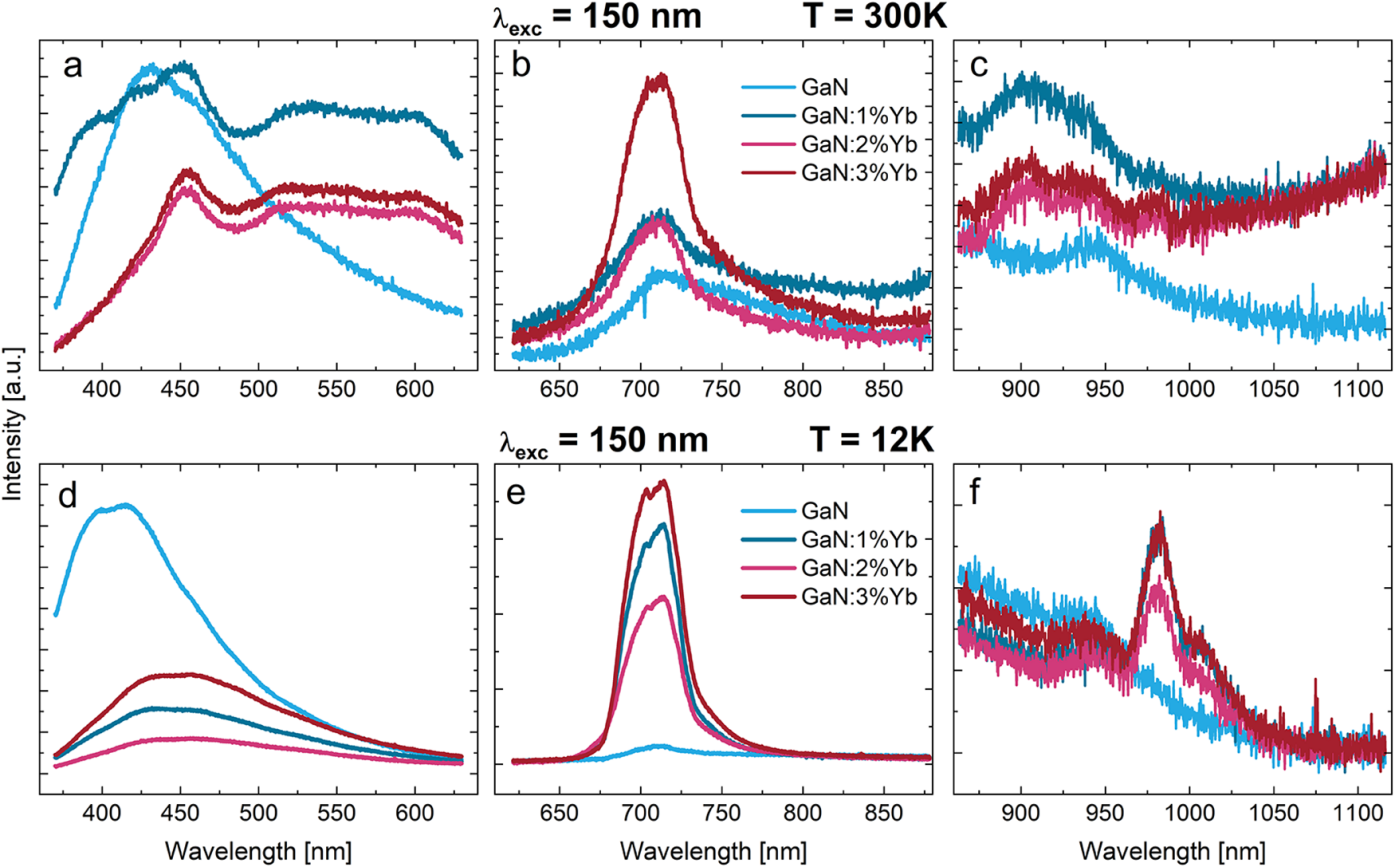
Vacuum ultraviolet (UV) emission spectra of GaN:Yb^3+^ nanopowders. Excitation λ = 150 nm. Emission in the visible-near-infrared range at 300 K (a–c) and at 12 K (d–f).

### Laser-Induced White Emission Spectra

The broad band laser-induced white emission of nanoceramic GaN doped with Yb^3+^ ions was measured by using a 975 nm CW laser diode as an excitation source. The measurements were conducted in a dynamic vacuum (10^–5^ mbar) as a function of power density, and the influence of atmospheric gases was excluded, ensuring the stability of the phenomenon being studied. The spectra are presented in Figure 6.

**Figure 6:**
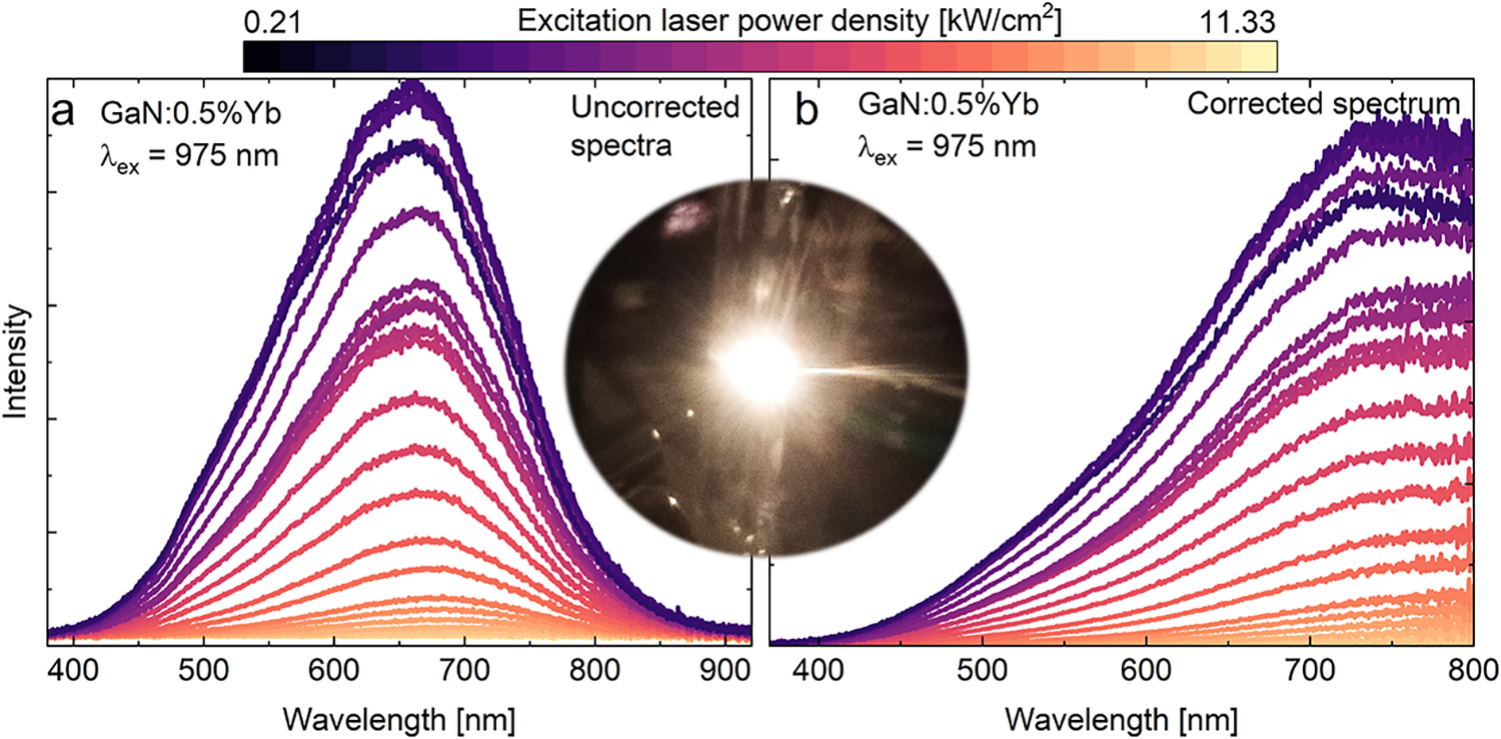
White light emission intensity as a function of laser power density [kW/cm^2^] using a CW laser 975 nm for GaN:0.5% Yb^3+^ nanoceramics. Uncorrected spectrum (a) and corrected (b). All spectra are in Supporting Information (S4).

The emission covers the visible region from 400 to 900 nm. The shape of the spectra is influenced by corrections applied to the detector sensitivity and the type of filter. The emission shape is similar to that observed in previous LIWE studies with other matrices.^18,33^ In contrast to conventional luminescence, the observed LIWE exhibits a characteristic threshold behavior (*P*_th_), where emission is initiated only after a specific excitation power density is exceeded. Experimental data indicate that *P*_th_ varies depending on the Yb^3+^ doping concentration (1.39–2.17 kW/cm^2^), which suggests that the dopant ions actively modify the effective absorption cross section of the material. The dependence of emission intensity *I*_LIWE_ on excitation laser power is characterized by the power law relation:

1 $$I_{LIWE}\left(P\right)\propto P^{N}$$

where *I*_LIWE_(*P*) is the intensity of emission, *P* is the excitation laser power density, and *N* represents the order of nonlinearity, related to the number of photons involved in multiphoton ionization responsible for laser-induced white emission.^21^ A crucial finding is the dependence of the nonlinearity parameter *N* on the Yb^3+^ dopant concentration presented in Figure 7. While undoped GaN exhibits a value of approximately 2.4, doped samples reach significantly higher values of up to 3.7. Considering that the photon energy of the 975 nm laser is ∼1.27 eV and the bandgap of GaN is ∼3.4 eV, a three-photon process is energetically required to bridge the bandgap. The presence of Yb^3+^ ions provides additional relaxation channels and increases the effective absorption cross section through these intermediate levels, which explains the qualitative change in the *N* parameter with increasing dopant concentration. This suggests that Yb ions play an active role in the LIWE mechanism, likely by introducing additional intermediate energy levels that facilitate multiphoton absorption. The process is more efficient but also more sensitive to the pump power. Thus, at high power densities, the emission intensity stops increasing, reaching a saturation plateau. This behavior can be attributed to the depletion of available ground-state electrons or thermal quenching processes.

**Figure 7:**
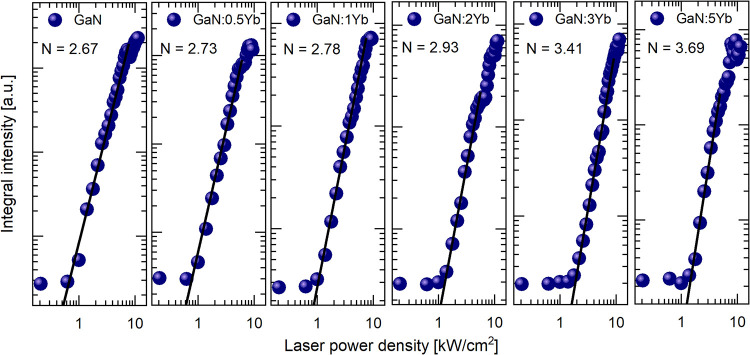
Integrated emission intensity (LIWE) as a function of excitation power density (log–log scale) under CW laser 975 nm excitation for GaN sample (a) and GaN-doped Yb^3+^ ions (b–f) nanoceramics. The line represents the exponential fit of *I = P* ^*N*^, and the value of nonlinearity (*N*) is given for the samples.

Furthermore, LIWE is a surface phenomenon dependent on the excitation laser spot size, as repeatedly indicated in previous studies.^35–37^ The process is highly sensitive to the sample surface and morphology. Consequently, the white emission may not be uniform across the entire investigated area, particularly under repeated exposure to high laser powers. Thermal effects also play a significant role; local heating induced by the focused laser beam can lead to grain growth, thereby reducing emission efficiency and causing partial degradation of the sample.

### Resistance Response

The dependence of photoelectric conductivity of GaN:Yb^3+^ nanoceramics on excitation laser power and applied voltage was investigated under focused laser beam (CW 975 nm) illumination. The measurements were performed at different applied voltages: 5, 10, 25, 50, and 150 V in the laser power range of 0.4–3.2 W. The results are shown in Figures 8, S3, and S4. To minimize the temperature factor and to evaluate the kinetics of photoresponse, the measurements were conducted in 60 s on/off cycles. It was observed that the dark resistance increased with the applied voltage. It suggests the non-Ohmic behavior, which is characteristic of polycrystalline materials, where grain boundaries can create potential barriers for carrier transport. This phenomenon is characteristic of granular semiconductors, where the transport is limited by thermionic emission or tunneling through intergranular potential barriers.^38,39^ However, upon turning on the laser and reaching the power threshold, the resistance instantly decreases by even several orders of magnitude. The determined photoconductivity threshold values averaged at 2.94 kW/cm^2^.

**Figure 8:**
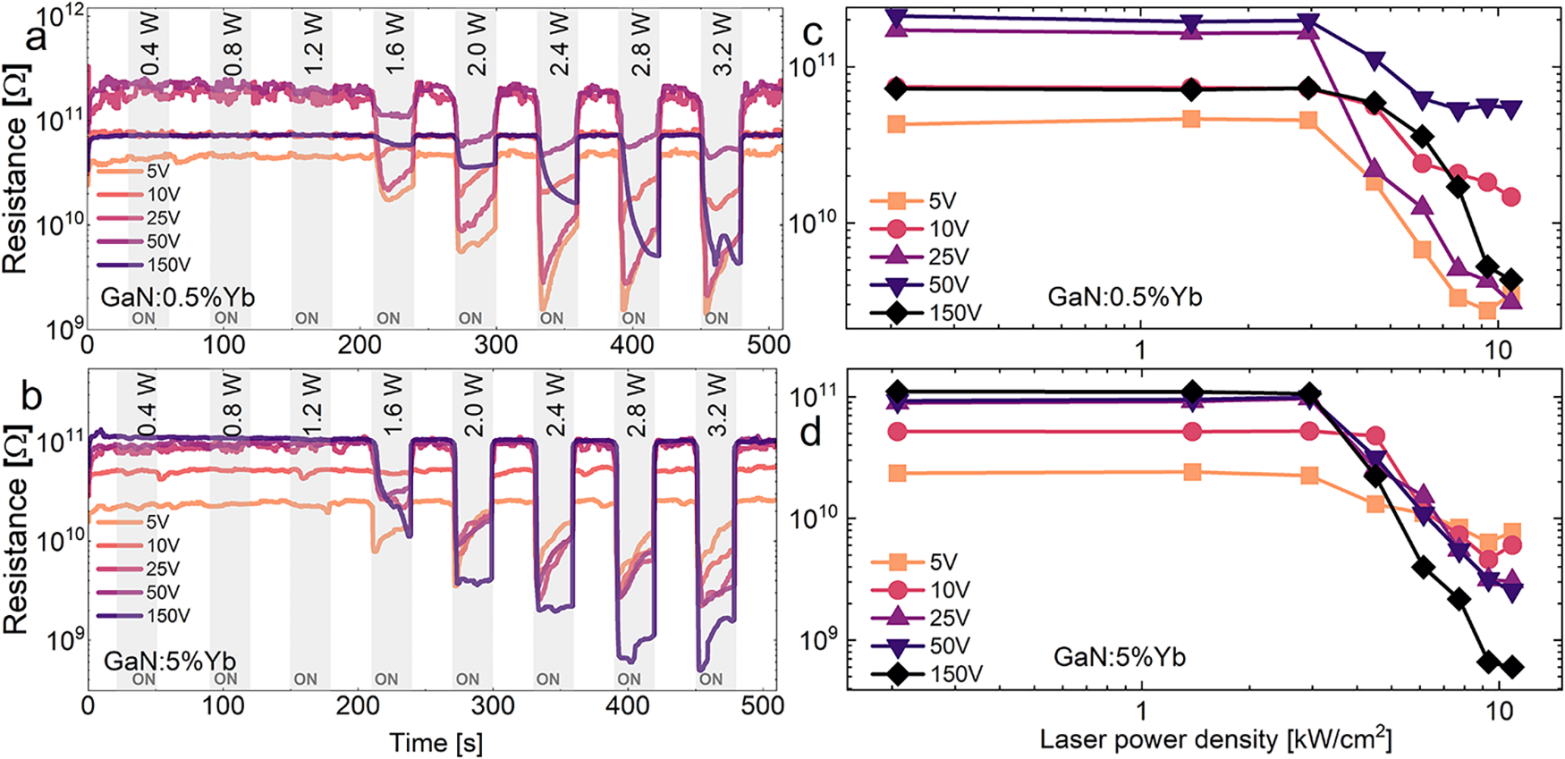
Photoresistance response as a function of time for GaN:0.5% Yb (a) and GaN:5% Yb (b) nanoceramics. Measurements were performed under CW laser excitation at 975 nm in 60 s on/off cycles. The dependence of resistance on laser power density is plotted in a log–log scale for various applied bias voltages (5–150 V) for the corresponding concentrations of Yb^3+^ ions (c, d). The graphs present only two concentrations for clarity; other measurements are in the SI.

Comparing these values to the white emission threshold values (1.39 and 2.17 kW/cm^2^), it can be concluded that higher power densities are required to trigger the photoresistance than for LIWE.

The difference in *P*_th_ suggests that the photocurrent and LIWE process are competing relaxation channels originating from the same multiphoton ionization process. This difference in thresholds is because emission can occur locally, while current must flow across the entire nanoceramic structure. This may be because recombination centers are activated at low power densities (responsible for LIWE), but the formation of a conductive flow path across grain boundaries requires a higher concentration of nonequilibrium carrier density to saturate the deep trap states.^23,40^ A decrease in resistance with increasing laser power is evident and proportional to the photocurrent. The most significant changes in resistance were observed at 150 V. The irregular fluctuations observed at lower excitation may be attributed to the surface phenomena, specifically the local topography and microscopic spot area of the focused laser beam. Furthermore, the local heating of the samples during the experiment causes changes in resistance. The presence of surface defects and material inhomogeneity can influence the signal stability.^41^

As a strong, persistent photocurrent (PPC) was observed, kinetic calculations were performed. The process is manifested by a slow return of the resistance to dark resistance after the laser is turned off. This behavior is related to the spatial separation of generated electrons and holes, induced by the electric fields at the grain boundaries, which inhibits direct recombination.^23,41^ To quantify this process, the photocurrent decay kinetics were analyzed using a biexponential function:

2 $$I\left(t\right)=I_{0}+A_{1}\;\exp\left(-\frac{t}{\tau_{1}}\right)+A_{2}\;\exp\left(-\frac{t}{\tau_{2}}\right)$$

where is τ_1_ fast decay time constant and τ_2_ is the slow decay time constant. The fitted parameters are presented in representative Table 1 and the Supporting Information (Tables S1–S6). The kinetic analysis reveals two distinct relaxation mechanisms. The fast component τ_1_ in the millisecond range corresponds to direct, fast recombination of excess carriers within the quasi-neutral bulk regions of the nanocrystallites from the valence band to the conduction band or recombination via shallow impurity centers, whereas the slow component τ_2_ ranging in seconds dominates the relaxation process. It is associated with the thermally activated release of carriers from deep trap states caused by structural defects, e.g., V_Ga_ located at the grain boundaries.^30,40^ The obtained samples exhibit high nanocrystallinity, as confirmed by powder diffraction. However, the presence of two decay times components indicates that the material has an optically active defect structure. This is because nanoceramics were used for photoconductivity measurements, where grain boundaries are traps that retain electrons, hence, the strong long-time component (τ_2_).

**Table 1:** Kinetic Parameters of Photocurrent Decay for GaN:Yb Nanoceramics Fitted with a Biexponential Function^a^

Sample	**τ**_**1**_
GaN	0.36
GaN:0.5% Yb	0.33
GaN:1% Yb	2.33
GaN:2% Yb	1.23
GaN:3% Yb	1.50
GaN:5% Yb	2.13

a Data presented for fixed bias voltage *U* = 150 V and measured at different laser power excitation power *P* = 2.8 W.

The voltage dependence of the slow decay time component τ_2_ was plotted (Figure S6). The analysis of τ_2_ provides key insights into the carrier mechanism. Above 25 V, where the electric field is stronger, a carrier sweep-out effect is observed for all single-phase samples (0–3% Yb^3+^ content). This suggests that under a stronger electric field, the Poole-Frenkel effect occurs. The effective ionization energy of the traps is lowered, accelerating the emission of released carriers and their drift toward the electrodes^38^

In the kinetic analysis of the obtained samples, the dependence related to Yb^3+^ doping plays a significant role. The GaN:3% Yb^3+^ sample represents a critical ytterbium ion concentration, corresponding to the solubility limit of the ions that can be incorporated into the GaN structure. XRD analysis confirms complete incorporation, but the crystal lattice strain is already very pronounced. Consequently, the sample exhibits a high density of defects acting as deep traps.^6^ Thus, relaxation times are prolonged at low voltages. However, upon application of a high electric field (150 V), the trap potential is effectively overcome, causing a substantial reduction of the τ_2_ constant to a low value (Figure 9a). In contrast to the effects described above for Yb-doped samples, the sample with a 5% Yb^3+^ content exhibits anomalous kinetics. The decay time τ_2_ remains constant or shows a nonmonotonic dependence on the applied voltage. This is related to phase segregation, which was also confirmed by XRD studies. At this level of Yb^3+^, the excess lanthanide ions are located at the grain boundaries.^6^ Such hetero phases are insulating and introduce structural disorder, creating additional deep interface states. This makes it difficult for carriers to flow by introducing competing retrapping and scattering processes. Consequently, carrier sweep-out by the external electric field is less effective compared to single-phase samples.^39^

**Figure 9:**
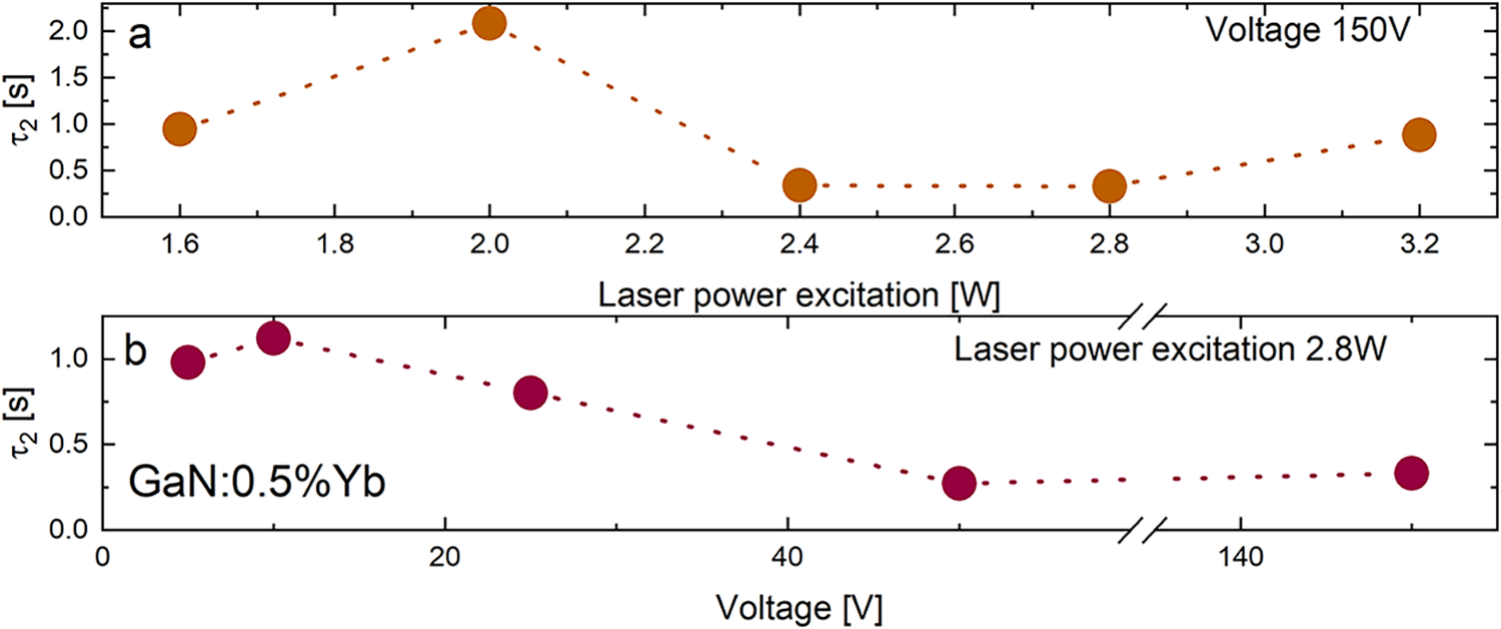
Dependence of the slow decay time τ_2_ on excitation laser power measured at constant bias voltage 5–150 V (a) and on applied bias voltage measured at different laser power excitation (b). The results are only for one concentration of 0.5% Yb^3+^ nanoceramics. All results are presented in the Supporting Information (Figures S6 and S7).

To further analyze the τ_2_ dependence, the decay time was plotted as a function of excitation laser power and is presented in Figures 9 and S7. The τ_2_ decay time component generally decreases with an increase in excitation power for samples with a pure structural phase. Gradual saturation of deep trap states is possible, shifting the quasi-Fermi level of electrons toward the conduction band edge. Once these centers are filled, the remaining carriers are forced to recombine through faster channels, shortening the effective lifetime.^23,38^ For the multiphase sample (GaN:5% Yb^3+^), no significant saturation was observed. The presence of the Ga_2_O_3_ phase likely creates a dense distribution of interphase traps, preventing saturation even at high excitation powers.^6^

As with broad band white light emission, the observed photoresistance follows a power law:

3 $$I_{Pc}\propto P^{N_{Pc}}$$

The *P*_c_ values are close to the nonlinearity for LIWE, indicating that both processes share a common source: multiphoton ionization. The simultaneous occurrence of LIWE and photocurrent suggests a coupled mechanism in which multiphoton absorption is associated with the generation of nonequilibrium carriers, which was also confirmed in a previous article about GaN.^42^ These carriers are then transferred through two channels via radiative recombination for LIWE and charge transport for the photocurrent. Importantly, the emission nonlinearity increases with the concentration of Yb^3+^ ions in the GaN matrix due to upconversion interactions, but the photocurrent nonlinearity dependent on these ions remains relatively stable. This probably means that the efficiency of the radiation path increases with the incorporation of Yb^3+^, but the free carrier rate responsible for the occurrence of photocurrent is related to the multiphoton absorption for the GaN matrix.^18,21,42^

To investigate the charge storage capacity of the material and the reversibility of the optical response, photoresistance measurements were performed in a continuous sweep mode of the excitation laser power at a constant bias voltage of 25 V (Figure 10). Two separate measurements were performed for each sample: one from low excitation laser power to high power (L → H → L) and from high power to low and back (H → L → H).

**Figure 10:**
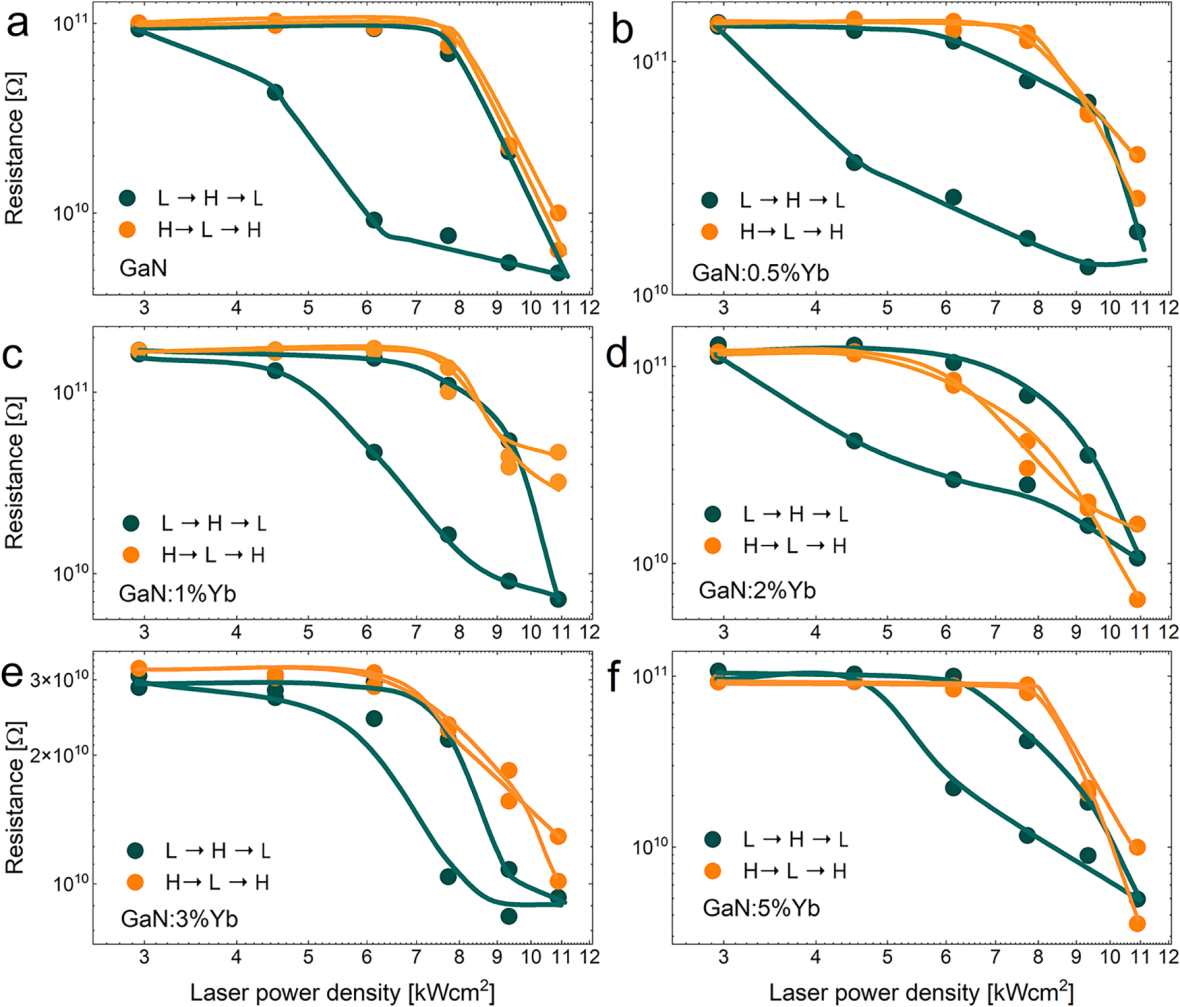
Optical memristive behavior of GaN:Yb^3+^ nanoceramics (a–f). The dependence of electrical resistance on excitation laser power density (CW 975 nm) measured at a constant bias voltage of 25 V in the power density range: 2.95–10.89 kW/cm^2^. L → H →L: from low excitation laser power to high power, and H → L → H: from high power to low and back.

The experimental results reveal two distinct behaviors of the process. A clear hysteresis loop is observed only in the low-power-high-power mode. In contrast, in the second mode, hysteresis is not visible; both paths overlap. The conclusion is that the appearance of hysteresis strongly depends on the initial state of the defect centers. The LHL measurement starts from a quasi-equilibrium state. The deep traps are not filled. As the laser power increases, some carriers are trapped by these traps, modifying the recombination rate until saturation is reached at maximum power. However, as the laser power is reduced again, the trapped carriers (PPC effect) slowly release, maintaining the material in a higher conductivity state, thus creating an opening of the loop.^23^ Therefore, if the measurement is performed from maximum laser power, then hysteresis is impossible because the deep trap states are already filled at the beginning. The material is effectively in an “on-memory” state.^24,43^ The calculated relaxation time τ_2_ is long, proving that the traps are occupied through the power change, and the return path remains unchanged due to the establishment of a balance between carrier generation and rapid recombination.

To quantitatively and physically describe the observed hysteresis effect as a memory effect, two parameters were introduced: optical hysteresis area (*S*_opt_) and hysteresis contrast (*H*_opt_).

The hysteresis loop area, *S*_opt_, was defined as the integral of the difference between the photoresistance during forward and backward measurements:

4 $$S_{\mathrm{opt}}=\int_{P_{\min}}^{P_{\max}}\left[R_{\mathrm{up}}\left(P\right)-R_{\mathrm{down}}\left(P\right)\right]\mathrm{dP}$$

where *R*_up_ is the resistance during the increase in laser power density and *R*_down_ is that during the decrease in laser power density. Furthermore, to account for the scale of permanent photoconductivity, an additional parameter, the Hysteresis Contrast (*H*_opt_), was defined:

5 $$H_{\mathrm{opt}}=\frac{S_{\mathrm{opt}}}{\int_{P_{\min}}^{P_{\max}}R_{\mathrm{down}}\left(P\right)\mathrm{dP}}\times 100\%$$

By introducing this index, we can easily normalize the loop size to the material conductivity. It provides information on the relative performance of the optical memory.^24,43^ The calculated parameters for the obtained samples are summarized in Table 2.

**Table 2:** Quantitative Analysis of the Optical Hysteresis Parameters for GaN:Yb^3+^ Nanoceramics^a^

sample	*S*_opt_ loop area [Ω·kW/cm^2^]	*H*_opt_ contrast [%]
GaN	3.47 × 10^11^	66.1
GaN:0.5% Yb	4.97 × 10^11^	64.8
GaN:1% Yb	4.56 × 10^11^	49.5
GaN:2% Yb	3.85 × 10^11^	58.5
GaN:3% Yb	3.44 × 10^10^	22.8
GaN:5% Yb	2.21 × 10^11^	42.4

a The parameters were calculated from the resistance-power characteristics measured in the low-to-high sweeping mode at a constant bias of 25 V.

A clear correlation is observed between the surface area of the hysteresis loop and the concentration of Yb^3+^ ions. The more Yb ions in the GaN matrix, the smaller the surface area. This behavior suggests that the undoped GaN material has the strongest “memory” effect, which dominates charge transport. Intrinsic defects at grain boundaries act as efficient charge storage centers, and their release dynamics are slow.^23,41^ Increasing the concentration of Yb^3+^ ions in the matrix changes the hysteresis surface area. The higher the concentration of Yb^3+^ dopant, the lower the *H*_opt_ value. The lowest value is 22.8% for the GaN:3% Yb^3+^ sample. The important role of the dopant is revealed, as the Yb^3+^ ions primarily allow for the regulation of deep traps. The reduction of *H*_opt_ to 22.8% proves that the Yb^3+^ ions can accelerate carrier relaxation. Despite the lattice strain, the recombination dynamics are the fastest, and intermediate energy levels effectively suppress the long-term memory effect compared to the pure GaN sample. As previously mentioned, this sample is at the interphase of Yb^3+^ ions incorporation into the GaN lattice, demonstrating the fastest carrier equilibrium in the valence and conduction bands.^4,11^ Interestingly, for the 5% Yb^3+^ sample, the hysteresis contrast increased again to 42.4%. This behavior correlates with the presence of additional phases, confirmed by the XRD method. The other phases at the grain boundaries lead to the formation of additional interphase traps. The defects introduced further increase the charge storage capacity.

In summary, a clear correlation was observed between the dopant concentration and the surface area of the resulting hysteresis. This demonstrates that the effect is related to the barrier potentials at the grain boundaries, and that these materials can function as an optically memristive system with programmable write capability.^24,43^

## Conclusions

In this work, nanocrystalline GaN:Yb^3+^ powders were successfully synthesized and subsequently consolidated into nanoceramics via a high-pressure, low-temperature (HPLT) sintering process. The conducted research included structural, spectroscopic, and optoelectronic characterization. The analysis of the obtained results leads to several significant conclusions.

Based on XRD measurements, the crystallization of the samples in the hexagonal wurtzite structure was confirmed. This method also verified the effective incorporation of Yb^3+^ ions into the GaN matrix up to a concentration limit of 3%, above this, phase segregation occurred, resulting in the precipitation of additional phases at the grain boundaries.

Spectroscopic studies revealed the defect-rich nature of the obtained materials. Bands originating directly from the host matrix, Yb^3+^ f–f transitions in the NIR range, as well as the active energy transfer between the matrix and the dopant were characterized. Synchrotron measurements proved to be particularly valuable, revealing an emission band at 700 nm attributed to deep defect traps.

Fundamental spectroscopic investigations enabled a better understanding and explanation of the second part of this study, specifically the characteristics of broad band white light emission as well as the photoresistive and optical response. A broad band in the visible region (LIWE) was observed under 975 nm laser excitation under vacuum conditions. This emission is characterized by threshold behavior and nonlinearity, which is a key indicator of a multiphoton ionization process. The stability of the emission in a vacuum confirms that the process involves charge carrier recombination, which eliminates pure thermal emission (blackbody radiation).

Furthermore, the white emission is strongly correlated to an increase in photoconductivity. This process is also nonlinear, exhibits threshold behavior, and is associated with multiphoton kinetics. Crucially, the analysis of decay times leads to the conclusion of slow relaxation dynamics and persistent photoconductivity (PPC). This confirms the hypothesis that the generated carriers are stored in deep traps, particularly at the grain boundaries.

The interplay between deep trapping and slow kinetics results in an interesting phenomenon, in the form of a hysteresis loop. The hysteresis contrast indicates that the conductivity is dominated by the memory of the optical history, and this effect appears to be fully controllable.

In summary, the investigated materials behave as a multifunctional optoelectronic memristive response. Grain boundary defects enable white light emission and induce slow relaxation, leading to charge storage, rendering these materials promising for memristive-like behavior devices and tunable white lighting applications.

## Supplementary Material



## Notes

SEM images, calculated band gap energy, resistance response, LIWE spectra, photocurrent decay times (PDF)
