# Supplementary material for: Yb3+-Doped GaN Nanoceramics as a New Material for Broad Band White Light Emission
Source: J Phys Chem C Nanomater Interfaces. 2026 Apr 8;130(16):6056–67. doi: 10.1021/acs.jpcc.6c00934 (PMC13308697; doi:10.1021/acs.jpcc.6c00934)
Supplement: Supplementary file 1 [file jp6c00934_si_001.pdf]

Supporting Information for Publication

Yb^{3+} doped GaN nanoceramics as a new material for broadband white light emission

A. Musiałek*, R. Tomala, G. E. Gagliardo Briuccia and W. Strek

Institute of Low Temperature and Structure Research,
Polish Academy of Sciences, 50422 Wroclaw, Poland

*Corresponding author

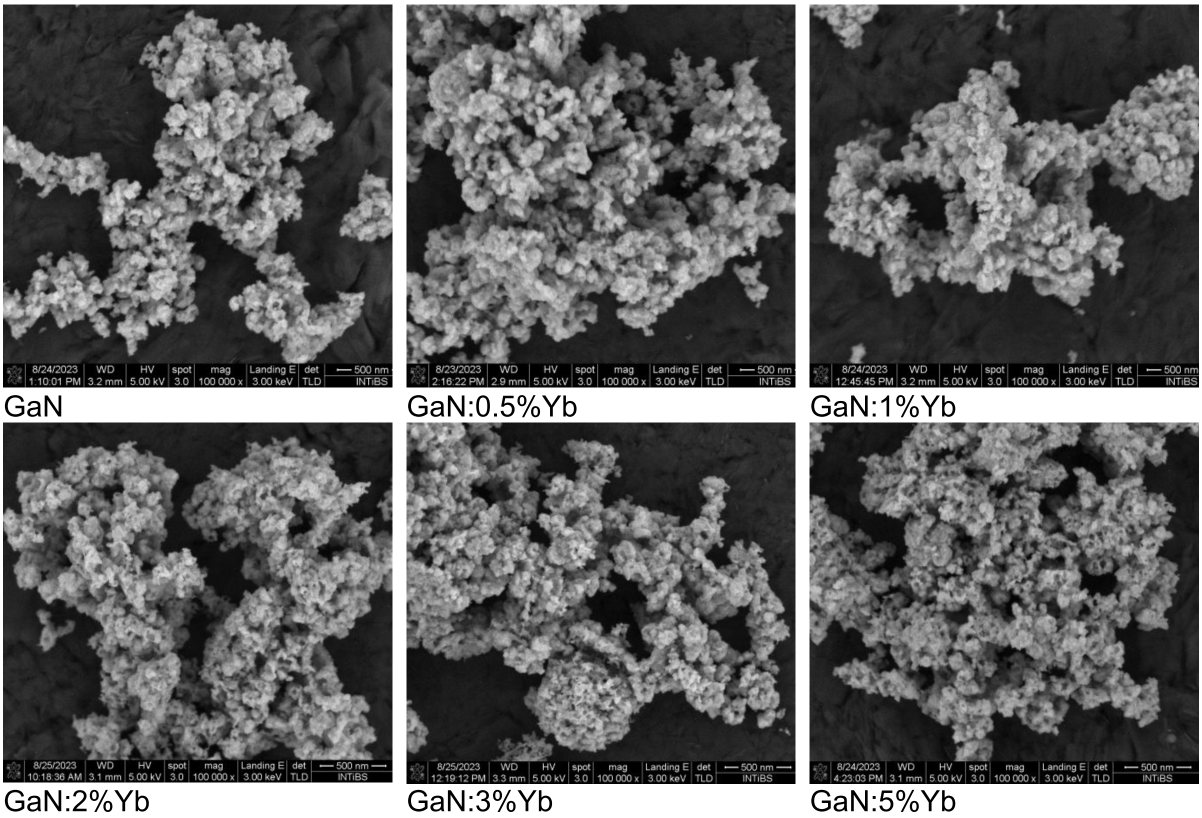

Fig. S1. SEM images of $Ga_{1-x}Yb_xN$ nanopowders.

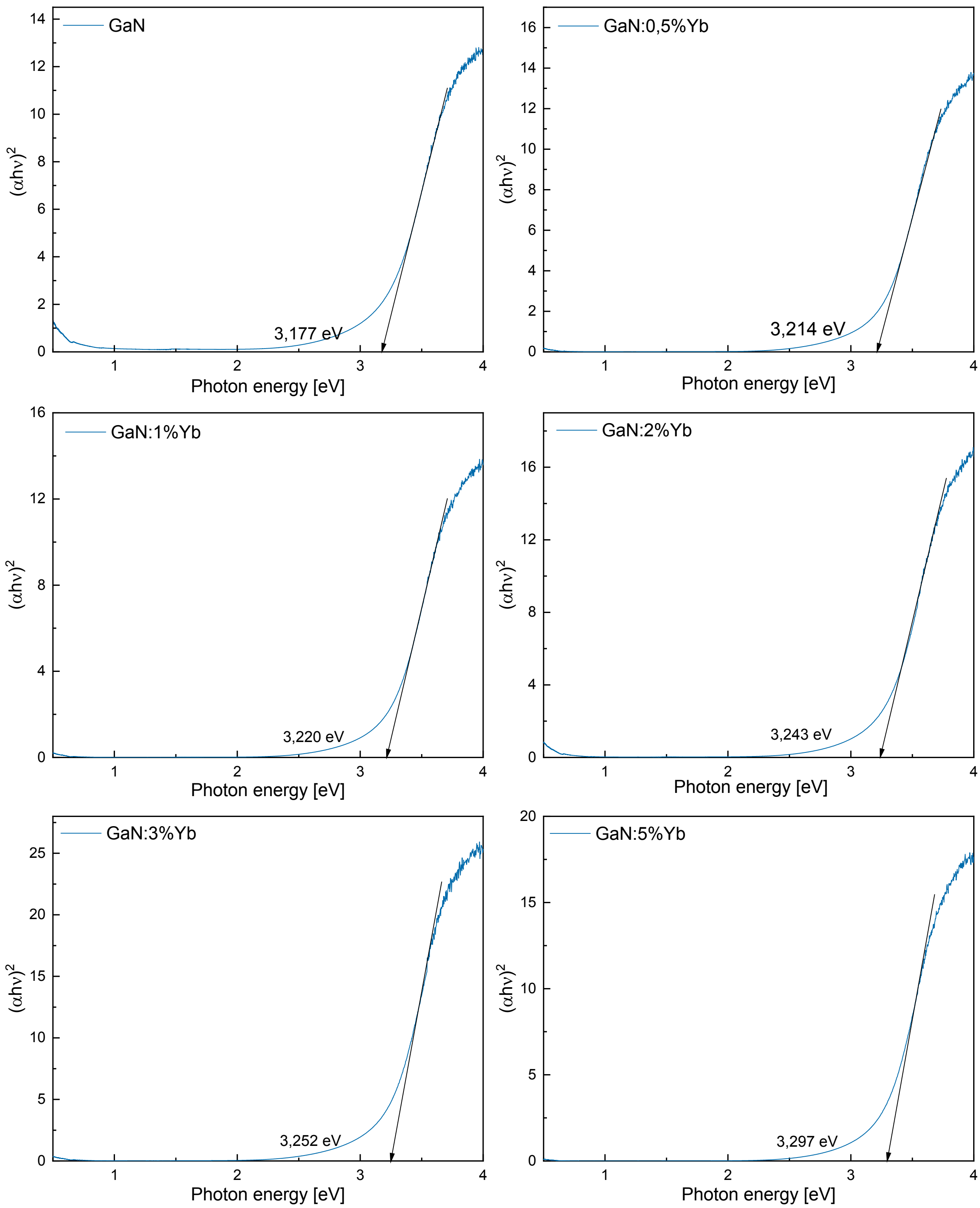

Fig. S2. Calculated band gap energy using Kubelka-Munk function for $Ga_{1-x}Yb_xN$ nanopowders.

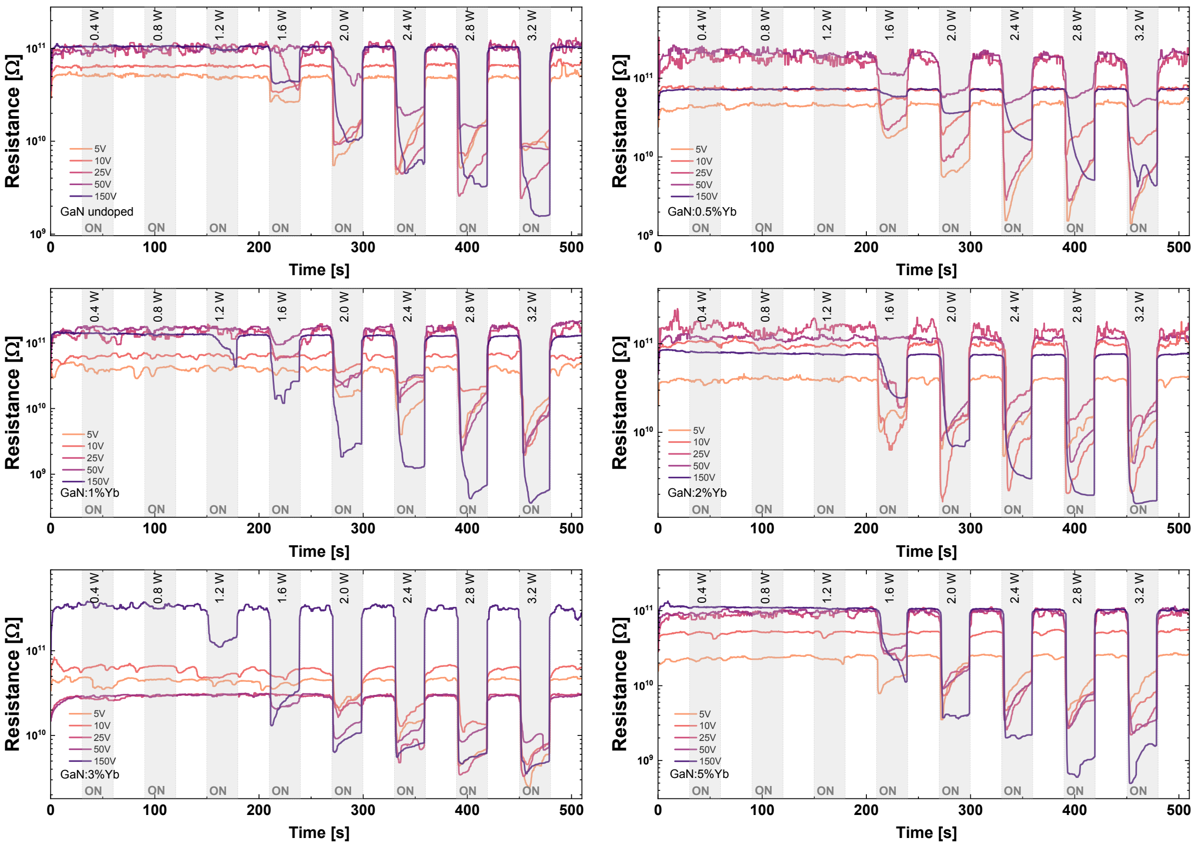

Fig. S3. Resistance response as a function of time for GaN (a) and GaN doped with Yb3+ ions (b-f). Measurements were performed under CW laser excitation at 975 nm in a 60s on/off cycles. The figures illustrate the dependence on: I. Increasing laser power, II. Different voltage biases.

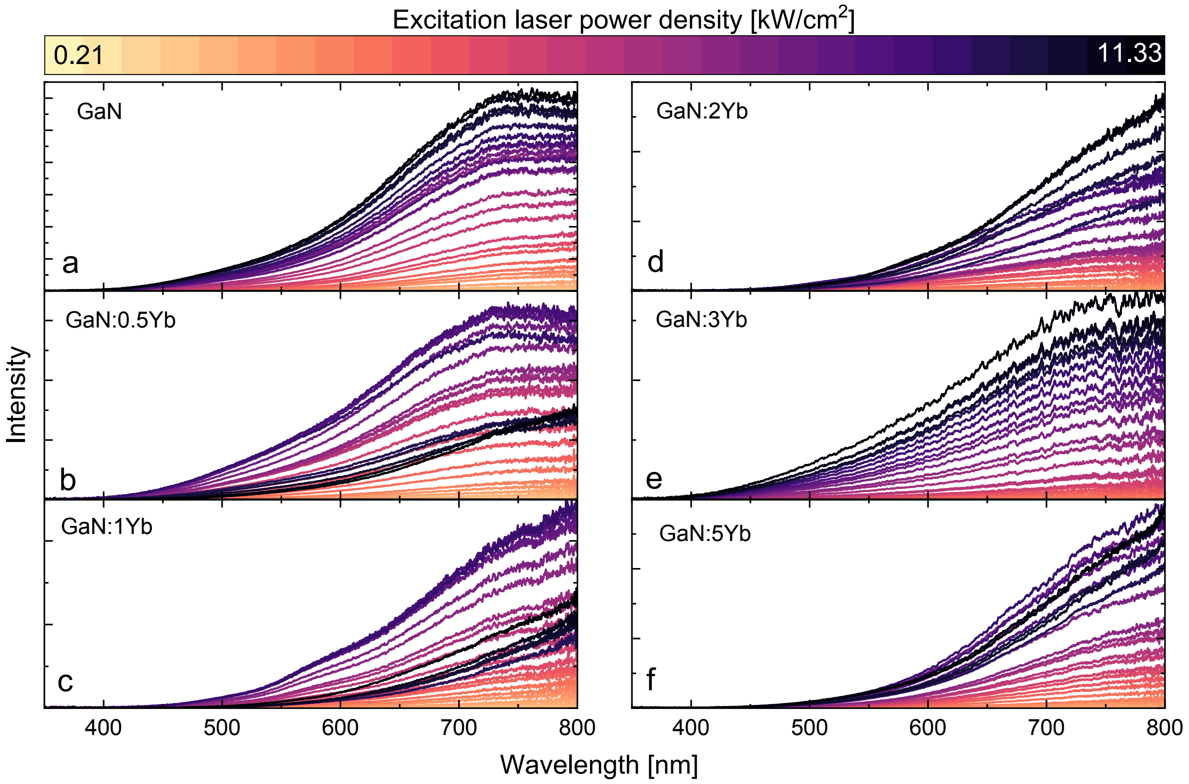

Fig. S4. White light emission intensity as a function of laser power density [kW/cm^2] using CW laser 975 nm for GaN (a) and GaN doped withYb^{3+} ions (b-f).

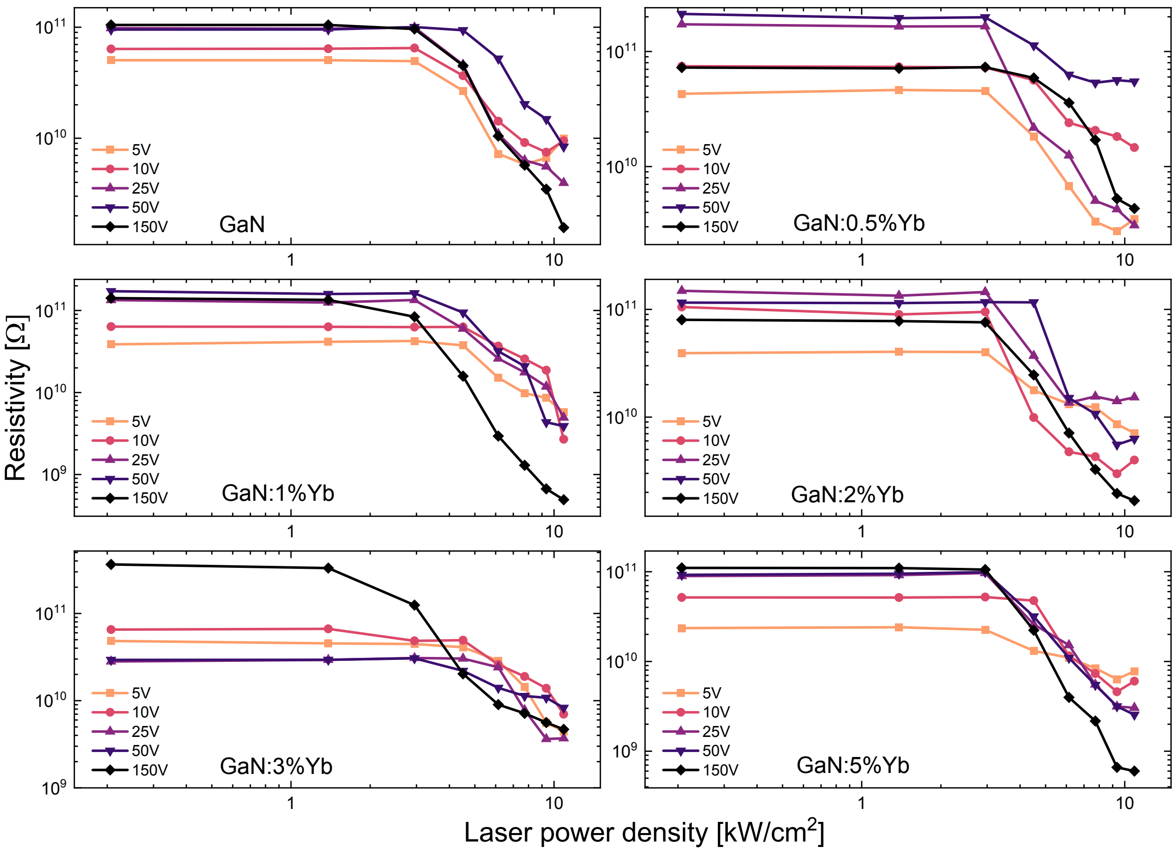

Fig. S5. Photoconductivity as a function of power laser in $Ga_{1-x}Yb_xN$ nanoceramics. The comparison of influence of different current field applied at the concentration of dopant concentration.

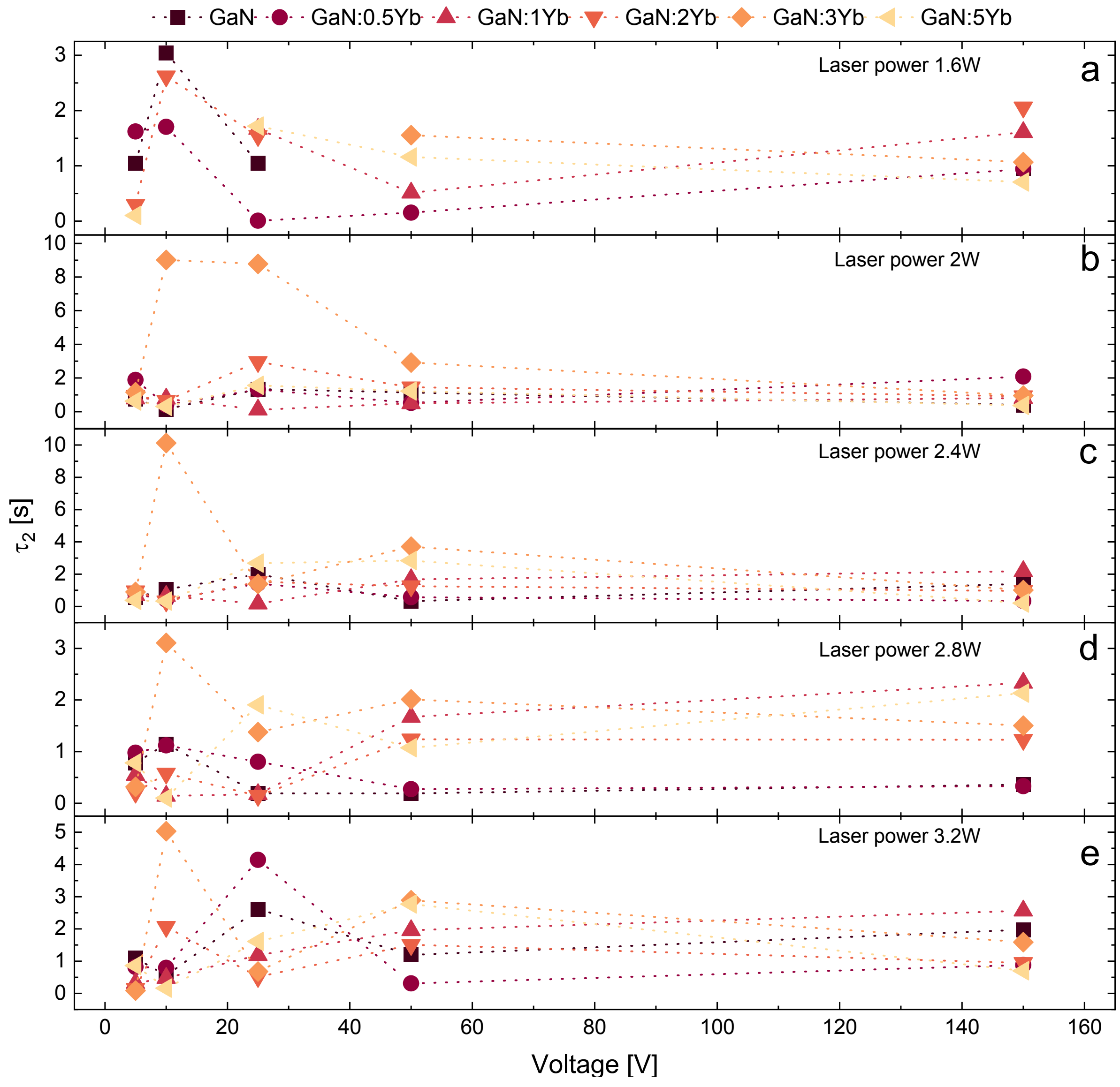

Fig. S6. Dependence of the slow decay time τ_2 on applied bias voltage measured at different laser power excitation 1.6 - 3.2 W (a-e).

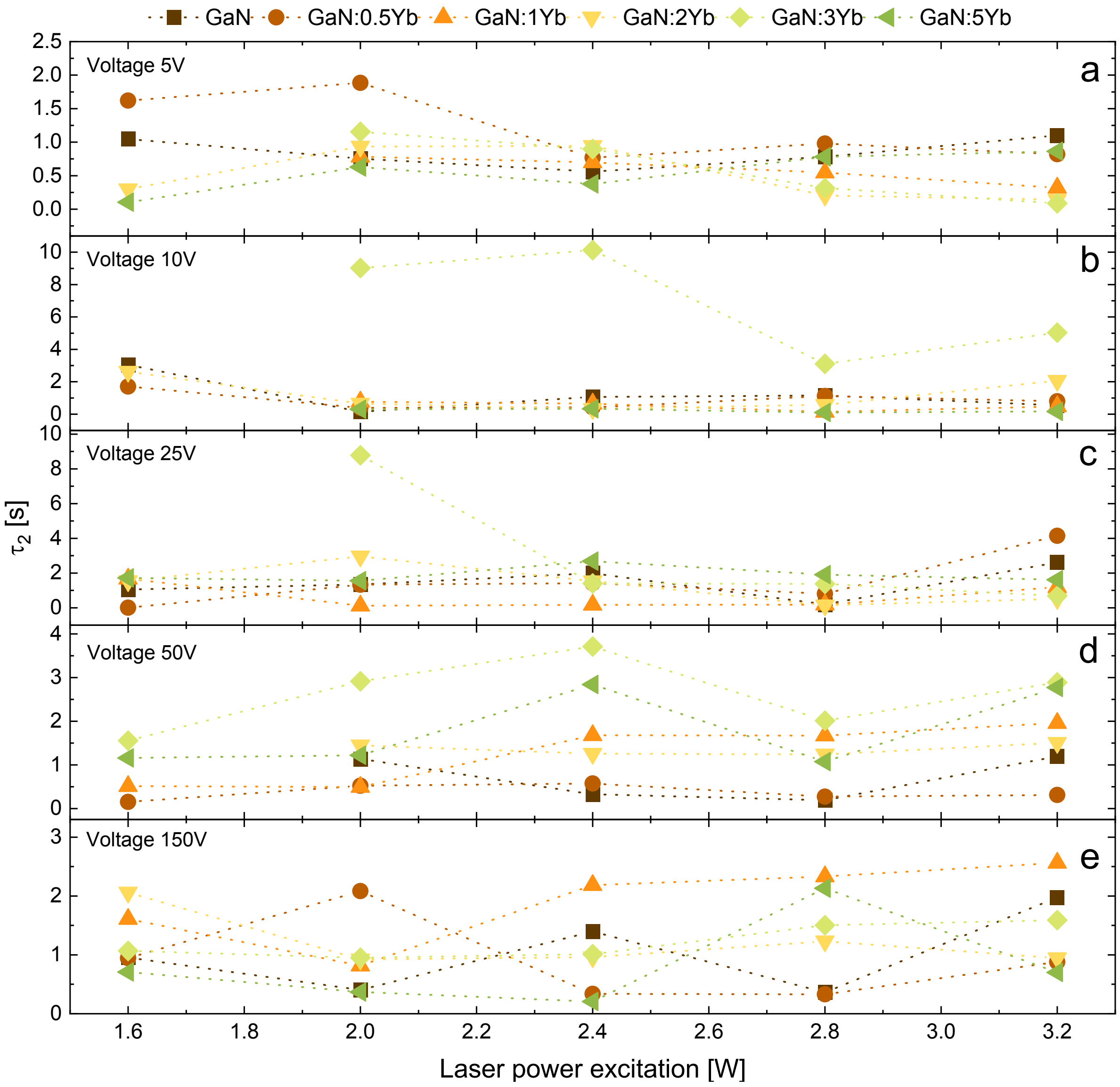

Fig. S7. Dependence of the slow decay time τ_2 on excitation laser power measured at constant bias voltage 5 - 150 V (a-e).

Table S1. Parameters of photocurrent decay times for nanoceramic GaN sample fitted with a bi-exponential function: $I(t) = I_o + A_1\exp(-t/\tau_1) + A_2\exp(-t/\tau_2)$. Measurements performed under 975 nm excitation.

**Sample**	**Voltage (V)**	**Power (W)**	**τ_1 (s)**	**A_1**	**τ_2 (s)**	**A_2**	**R^2**
**GaN**	5	1.60	0.271	- 3.78E+09	1.05	- 1.62E+10	0.99
		2.00	0.003	- 1.50E+10	0.76	- 1.65E+10	1.00
		2.40	0.003	- 5.32E+09	0.56	- 2.17E+10	0.98
		2.80	0.005	- 1.59E+10	0.78	- 1.40E+10	0.99

		3.20	0.092	-2.64E+10	1.10	-1.38E+10	0.99
	10	1.60	0.150	-1.99E+10	3.05	-4.49E+09	1.00
		2.00	0.003	-2.24E+10	0.14	-2.24E+10	0.99
		2.40	0.005	-2.32E+10	1.06	-1.94E+10	0.95
		2.80	0.003	-2.03E+10	1.15	-1.61E+10	0.90
		3.20	0.002	-2.84E+10	0.55	-2.06E+10	0.98
	25	1.60	0.004	-6.09E+09	1.05	-3.50E+10	0.99
		2.00	0.006	-4.11E+10	1.34	-3.07E+10	0.98
		2.40	0.005	-4.07E+10	1.99	-5.62E+10	0.94
		2.80	0.001	-2.51E+10	0.19	-2.40E+10	0.99
		3.20	0.022	-5.39E+14	2.61	-5.37E+10	0.97
	50	1.60	-	-	-	-	-
		2.00	0.005	-1.47E+10	1.14	-3.17E+10	0.98
		2.40	0.003	-3.27E+10	0.33	-3.21E+10	0.98
		2.80	0.004	-2.98E+10	0.19	-2.92E+10	0.99
		3.20	0.007	-4.04E+10	1.19	-6.24E+10	0.94
	150	1.60	0.005	-4.25E+10	0.96	-1.72E+10	0.98
		2.00	0.002	-4.56E+10	0.40	-4.07E+10	0.96
		2.40	0.053	-7.90E+10	1.40	-1.90E+10	1.00
		2.80	0.004	-4.83E+10	0.36	-4.28E+10	0.97
		3.20	0.111	-8.34E+10	1.97	-1.88E+10	1.00

Table S2. Parameters of photocurrent decay times for nanoceramic GaN:0.5%Yb sample fitted with a bi-exponential function: I(t) = I_o + A_1exp($-t/\tau_1$) + A_2exp($-t/\tau_2$). Measurements performed under 975 nm excitation.

**Sample**	**Voltage (V)**	**Power (W)**	**τ_1 (s)**	**A_1**	**τ_2 (s)**	**A_2**	**R^2**
**GaN:0.5Yb**	5	1.60	0.003	-9.34E+08	1.62	-2.75E+09	0.98
		2.00	0.185	-2.98E+10	1.88	-9.75E+09	0.99
		2.40	0.083	-2.45E+10	0.77	-1.15E+10	0.99
		2.80	0.005	-7.19E+09	0.98	-6.85E+09	0.96

		3.20	0.005	-2.14E+10	0.82	-1.83E+10	0.98
	10	1.60	0.269	-5.19E+09	1.71	-1.90E+09	0.99
		2.00	0.005	-1.07E+10	0.39	-2.09E+10	0.97
		2.40	0.082	-1.41E+10	0.42	-3.09E+10	1.00
		2.80	0.006	-1.81E+10	1.12	-2.84E+10	0.99
		3.20	0.005	-2.09E+10	0.79	-3.01E+10	0.98
	25	1.60	0.172	-4.22E+10	0.01	-4.30E+10	0.99
		2.00	0.145	-5.82E+10	1.32	-1.07E+11	0.98
		2.40	0.005	-4.46E+10	1.42	-1.10E+11	0.98
		2.80	0.762	2.67E+10	0.80	-1.06E+11	0.99
		3.20	0.202	-6.93E+10	4.14	-1.06E+11	0.96
	50	1.60	0.008	-5.27E+09	0.15	-5.04E+09	0.99
		2.00	0.009	-2.24E+10	0.52	-2.33E+10	0.88
		2.40	0.010	-9.46E+09	0.58	-9.09E+09	0.96
		2.80	0.004	-2.35E+10	0.27	-2.32E+10	0.99
		3.20	0.002	-2.45E+10	0.31	-2.29E+10	0.98
	150	1.60	0.020	-5.31E+09	0.94	-6.87E+09	0.92
		2.00	0.347	-3.35E+10	2.08	-2.68E+09	0.93
		2.40	0.004	-2.47E+10	0.34	-2.16E+10	0.95
		2.80	0.005	-3.35E+10	0.33	-2.87E+10	0.97
		3.20	0.002	-2.08E+10	0.88	-2.10E+10	0.82

Table S3. Parameters of photocurrent decay times for nanoceramic GaN:1%Yb sample fitted with a bi-exponential function: $I(t) = I_o + A_1\exp(-t/\tau_1) + A_2\exp(-t/\tau_2)$. Measurements performed under 975 nm excitation.

**Sample**	**Voltage (V)**	**Power (W)**	**τ_1 (s)**	**A_1**	**τ_2 (s)**	**A_2**	**R^2**
**GaN:1Yb**	5	1.60	-	-	-	-	-
		2.00	0.006	-8.60E+09	0.78	-7.82E+09	0.97
		2.40	0.004	-1.41E+10	0.70	-1.38E+10	0.99
		2.80	0.003	-3.58E+09	0.55	-2.12E+10	1.00
		3.20	0.004	-1.14E+10	0.32	-1.17E+10	0.93
	10	1.60	-	-	-	-	-
		2.00	0.025	-3.88E+13	0.77	-2.01E+09	0.99
		2.40	0.020	-1.45E+14	0.65	-1.19E+10	0.90
		2.80	0.034	1.51E+13	0.14	-2.20E+11	1.00
		3.20	0.031	-2.57E+13	0.48	-1.82E+10	1.00
	25	1.60	0.229	-8.26E+09	1.67	-2.75E+10	0.99

		2.00	0.001	-8.57E+09	0.12	-8.44E+09	0.99
		2.40	0.001	-1.30E+10	0.17	-1.19E+10	0.98
		2.80	0.005	-1.40E+10	0.17	-1.30E+10	0.99
		3.20	0.003	-3.85E+10	1.18	-7.93E+10	0.97
	50	1.60	0.005	-8.17E+09	0.51	-8.19E+09	0.89
		2.00	0.004	-2.98E+10	0.49	-2.99E+10	0.99
		2.40	0.002	-3.80E+10	1.68	-1.18E+11	0.95
		2.80	0.007	-6.66E+10	1.67	-1.04E+11	0.99
		3.20	0.006	-7.84E+10	1.96	-1.07E+11	0.96
	150	1.60	0.008	-5.81E+10	1.61	-3.25E+10	0.97
		2.00	0.001	-5.58E+10	0.82	-4.41E+10	0.93
		2.40	0.001	-8.87E+10	2.18	-3.30E+10	0.99
		2.80	0.076	-7.76E+10	2.33	-4.95E+10	1.00
		3.20	0.004	-6.05E+10	2.56	-6.07E+10	0.99

Table S4. Parameters of photocurrent decay times for nanoceramic GaN:2%Yb sample fitted with a bi-exponential function: $I(t) = I_o + A_1\exp(-t/\tau_1) + A_2\exp(-t/\tau_2)$. Measurements performed under 975 nm excitation.

**Sample**	**Voltage (V)**	**Power (W)**	**τ_1 (s)**	**A_1**	**τ_2 (s)**	**A_2**	**R^2**
**GaN:2Yb**	5	1.60	0.001	-5.78E+08	0.30	-5.58E+08	0.98
		2.00	0.091	-1.76E+10	0.94	-5.40E+09	0.99
		2.40	0.003	-1.04E+10	0.94	-8.32E+09	0.99
		2.80	0.005	-1.22E+10	0.21	-1.23E+10	0.98
		3.20	0.005	-1.24E+10	0.14	-1.24E+10	0.99
	10	1.60	0.113	-6.07E+10	2.62	-1.96E+10	0.97
		2.00	0.004	-3.42E+10	0.65	-3.40E+10	0.93
		2.40	0.005	-3.79E+10	0.32	-3.68E+10	0.99
		2.80	0.002	-4.06E+10	0.58	-4.59E+10	0.98

		3.20	0.068	-6.08E+10	2.07	-3.20E+10	0.99
	25	1.60	0.004	-2.92E+10	1.54	-6.19E+10	0.95
		2.00	0.001	-2.92E+10	2.96	-9.00E+10	0.95
		2.40	0.226	-2.74E+10	1.53	-4.87E+10	0.99
		2.80	0.006	-4.34E+09	0.14	-4.21E+09	0.99
		3.20	0.003	-3.25E+10	0.51	-3.54E+10	0.95
	50	1.60	-	-	-	-	-
		2.00	0.006	-4.81E+10	1.45	-4.80E+10	0.99
		2.40	0.005	-4.58E+10	1.26	-4.98E+10	0.92
		2.80	0.002	-9.16E+09	1.24	-1.13E+10	0.97
		3.20	0.116	-4.76E+10	1.51	-5.04E+10	0.98
	150	1.60	0.003	-1.85E+10	2.06	-1.17E+10	0.82
		2.00	0.004	-2.58E+10	0.92	-2.19E+10	0.87
		2.40	0.005	-3.90E+10	0.96	-1.94E+10	0.94
		2.80	0.006	-3.91E+10	1.23	-1.98E+10	0.94
		3.20	0.002	-3.68E+10	0.94	-2.21E+10	0.93

Table S5. Parameters of photocurrent decay times for nanoceramic GaN:3%Yb sample fitted with a bi-exponential function: $I(t) = I_o + A_1\exp(-t/\tau_1) + A_2\exp(-t/\tau_2)$. Measurements performed under 975 nm excitation.

**Sample**	**Voltage (V)**	**Power (W)**	**τ_1 (s)**	**A_1**	**τ_2 (s)**	**A_2**	**R^2**
**GaN:3Yb**	5	1.60	-	-	-	-	-
		2.00	0.005	-5.40E+09	1.16	-5.04E+09	0.92
		2.40	0.003	-1.43E+10	0.90	-1.07E+10	0.95
		2.80	0.002	-1.96E+10	0.32	-1.73E+10	0.98
		3.20	0.005	-1.94E+10	0.09	-1.93E+10	1.00
	10	1.60	-	-	-	-	-
		2.00	0.007	-9.09E+09	9.02	-2.41E+10	1.00
		2.40	0.081	-2.37E+10	10.12	-2.10E+10	0.98

		2.80	0.074	-3.31E+10	3.11	-1.41E+10	0.99
		3.20	0.006	-2.96E+10	5.03	-1.99E+10	0.98
	25	1.60	-	-	-	-	-
		2.00	0.861	-2.59E+09	8.78	-3.14E+09	0.93
		2.40	0.004	-1.31E+10	1.40	-8.19E+09	0.98
		2.80	0.005	1.18E+08	1.38	-5.31E+09	0.91
		3.20	0.222	-9.91E+09	0.68	-1.20E+10	0.98
	50	1.60	0.008	-1.04E+09	1.56	-4.55E+09	0.96
		2.00	0.040	-1.01E+10	2.92	-4.03E+09	0.99
		2.40	0.079	-1.02E+10	3.71	-3.91E+09	0.97
		2.80	0.007	-7.92E+09	2.01	-5.78E+09	0.95
		3.20	0.064	-1.80E+10	2.89	-3.48E+09	1.00
	150	1.60	0.004	-1.61E+11	1.07	-1.34E+11	0.99
		2.00	0.001	-1.57E+11	0.95	-1.25E+11	0.98
		2.40	0.003	-1.65E+11	1.01	-1.17E+11	0.96
		2.80	0.005	-1.64E+11	1.50	-1.40E+11	0.86
		3.20	0.080	-2.29E+11	1.59	-5.28E+10	1.00

Table S6. Parameters of photocurrent decay times for nanoceramic GaN:5%Yb sample fitted with a bi-exponential function: $I(t) = I_o + A_1\exp(-t/\tau_1) + A_2\exp(-t/\tau_2)$. Measurements performed under 975 nm excitation.

**Sample**	**Voltage (V)**	**Power (W)**	**τ_1 (s)**	**A_1**	**τ_2 (s)**	**A_2**	**R^2**
**GaN:5Yb**	5	1.6	0.00824	-5.12E+09	0.10323	-5.12E+09	1.00
		2.0	0.00371	-1.71E+09	0.62757	-1.70E+09	0.93
		2.4	0.00162	-4.48E+09	0.37585	-3.35E+09	1.00
		2.8	0.00162	-6.26E+09	0.78023	-4.10E+09	0.94
		3.2	0.00501	-4.19E+09	0.86327	-3.56E+09	0.96
	10	1.6	-	-	-	-	-
		2.0	0.00473	-1.35E+10	0.30491	-1.20E+10	0.96

		2.4	0.00373	-1.85E+10	0.32604	-1.64E+10	0.95
		2.8	0.00327	-2.09E+10	0.09911	-2.08E+10	0.99
		3.2	0.00446	-2.16E+10	0.16196	-2.17E+10	0.98
	25	1.6	0.00182	-1.54E+10	1.71855	-5.73E+10	0.89
		2.0	0.00439	-2.04E+10	1.55598	-5.63E+10	0.91
		2.4	0.00216	-1.55E+10	2.6798	-9.27E+10	0.94
		2.8	0.00129	-3.60E+10	1.90573	-6.77E+10	0.92
		3.2	0.00775	-4.56E+10	1.60902	-3.41E+10	0.99
	50	1.6	0.00408	-2.43E+10	1.15966	-3.70E+10	0.96
		2.0	0.00347	-4.30E+10	1.22078	-3.68E+10	0.96
		2.4	0.37462	-6.85E+10	2.84026	-3.53E+10	0.92
		2.8	0.00457	-4.88E+10	1.07297	-4.26E+10	0.97
		3.2	0.29464	-7.63E+10	2.77208	-3.27E+10	0.95
	150	1.6	0.00599	-3.78E+10	0.70834	-3.60E+10	0.92
		2.0	0.00551	-5.13E+10	0.36956	-4.50E+10	0.98
		2.4	0.00181	-4.97E+10	0.2079	-4.94E+10	0.97
		2.8	0.05366	-8.50E+10	2.13188	-1.69E+10	1.00
		3.2	0.00435	-4.01E+10	0.70183	-3.96E+10	0.94
